# A Wearable AI‐Driven Mask with Humidity‐Sensing Respiratory Microphone for Non‐Vocal Communication

**DOI:** 10.1002/advs.202504343

**Published:** 2025-06-27

**Authors:** Jianfei Wang, Hongyu Zhang, Xiaomin Wu, Mingyan Gao, He Wen, Zhibo Zhang, Kremena Makasheva, Wen Jung Li, Zuobin Wang

**Affiliations:** ^1^ International Research Centre for Nano Handling and Manufacturing of China Changchun University of Science and Technology Jilin 130022 China; ^2^ CAS‐CityU Joint Laboratory for Robotic Research Department of Mechanical Engineering City University of Hong Kong Kowloon Hong Kong 999077 China; ^3^ College of Medical Informatics Chongqing Medical University Chongqing 400016 China; ^4^ Centre for Opto/Bio‐Nano Measurement and Manufacturing Zhongshan Institute of Changchun University of Science and Technology Zhongshan 528437 China; ^5^ Laboratory on Plasma and Conversion of Energy (LAPLACE) CNRS Toulouse 31062 France

**Keywords:** contactless sensing, gold nanoparticles, human‐machine interactions, humidity sensor, respiration language

## Abstract

Hoarseness and dysphonia caused by vocal cord conditions or laryngeal surgeries significantly hinder communication and quality of life. This study presents a plug‐and‐play humidity‐sensing respiratory microphone (HSRM) with generalized features for individual users. Leveraging gold nanoparticle‐based humidity sensors integrated into commercially available wearable face masks, the system enables patients to produce verbal communication without relying on vocal cord activity. By integrating nanoparticle‐enhanced humidity sensors with advanced convolutional neural networks, the HSRM system accurately decodes respiratory patterns into intelligible speech, achieving a recognition accuracy of 85.61%. Leveraging nanoparticle‐polymer interfaces that effectively convert atmospheric humidity fluctuations into precise electrical signals, the system pioneers a contactless and non‐invasive paradigm in assistive speech technology. This innovation addresses limitations of existing devices, such as reliance on residual vocal fold vibrations or skin‐contact sensors, offering a practical generalized solution for individuals with aphonia. With its potential to facilitate naturalistic communication and transform healthcare applications, the HSRM system sets a new benchmark in wearable assistive technologies for voice rehabilitation and human‐machine interaction.

## Graphical abstract

A wearable, plug‐and‐play respiratory microphone—featuring a gold nanoparticle‐based humidity sensor—facilitates voice‐free verbal communication for patients with impaired vocal fold function.

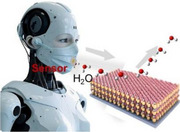

## 1 Introduction

Voice represents one of the fundamental modalities of human communication, enabling individuals to articulate thoughts, emotions, and information. Concurrently, sound serves as the medium through which language is conveyed, with traditional perspectives asserting that language cannot be perceived or comprehended without sound.^[^ ^1^ ^]^ The importance of the human voice as a communicative instrument is extensively documented in the scholarly literature.^[^ ^2^, ^3^ ^]^ Nevertheless, in the context of contemporary society's incessant activity, our voices are confronted with unprecedented challenges. Various factors, including noise pollution, medical conditions, accidents, and the aging process, pose latent threats that gradually undermine vocal health. Conditions such as vocal nodules,^[^ ^4^ ^]^ vocal polyps, laryngeal tumors,^[^ ^5^ ^]^ neuromuscular disorders,^[^ ^6^, ^7^ ^]^ vocal cord paralysis,^[^ ^8^ ^]^ chronic laryngitis,^[^ ^9^ ^]^ allergic reactions,^[^ ^10^ ^]^ smoking,^[^ ^11^ ^]^ and excessive vocal cord use not only impair individuals' capacity to vocalize but also significantly hinder their social interactions and emotional expression.^[^ ^12^, ^13^ ^]^ Despite the considerable advancements in modern medical technology regarding the early diagnosis, treatment, and rehabilitation of vocal cord disorders,^[^ ^14^, ^15^ ^]^ which encompass minimally invasive surgical techniques,^[^ ^16^ ^]^ pharmacological interventions, and physiotherapy,^[^ ^17^ ^]^ patients frequently endure a protracted and arduous rehabilitation process.^[^ ^18^ ^]^ During this phase, individuals encounter obstacles to effective communication with their external environment, which exacerbates their psychological distress and restricts their engagement in social activities. Existing assistive technologies such as electromyography (EMG) interfaces,^[^ ^19^ ^]^ piezoelectric throat microphones,^[^ ^20^ ^]^ and subvocal air‐pressure sensors,^[^ ^21^ ^]^ remain constrained by several critical limitations: 1) dependence on residual vocal fold vibrations or muscle contractions, rendering them ineffective for patients with complete glottal insufficiency;^[^ ^22^ ^]^ 2) susceptibility to ambient acoustic interference;^[^ ^23^ ^]^ 3) ergonomic challenges from skin‐contact electrodes causing dermatological complications in long‐term users.^[^ ^24^ ^]^

Therefore, the development of an innovative wearable medical device designed to facilitate communication during the treatment and rehabilitation of patients with vocal cord impairments is of paramount importance.^[^ ^25^ ^]^ To develop wearable throat sensors, numerous researchers have progressively advanced the design of high‐quality sensors utilizing nanomaterials such as polyvinylidene fluoride,^[^ ^26^ ^]^ carbon nanotube,^[^ ^27^ ^]^ nanowire,^[^ ^3^ ^]^ MoS_2_,^[^ ^28^ ^]^ graphene,^[^ ^29^, ^30^ ^]^ ZnO,^[^ ^31^ ^]^ and MXene.^[^ ^32^ ^]^ The majority of laryngeal sensors operate based on piezoelectric,^[^ ^33^ ^]^ capacitive,^[^ ^34^ ^]^ and self‐powered mechanisms with the potential to recognize speech signals.^[^ ^35^, ^36^ ^]^ Despite the significant progress achieved in the domain of wearable laryngeal sensor technology, most throat sensors predominantly depend on external sound pressure and vibrational stimuli, necessitating a tight fit within the larynx to effectively capture subtle vibrations in the laryngeal muscles.^[^ ^37^ ^]^ This methodology is particularly ineffective for patients with compromised vocal cords as their vocal cords may lack the capacity to generate sufficient vibrations to activate conventional sensors, thereby constraining the applicability and efficacy of these devices.^[^ ^38^ ^]^

To overcome the above barriers, we present a plug‐and‐play humidity‐sensing respiratory microphone (HSRM) that fundamentally redefines non‐vocal communication through the following three paradigm‐shifting innovations. 1) Non‐contact biosignal transduction: leveraging humidity gradients generated by exhaled breath rather than mechanical vibrations, enabling speech without laryngeal contact. 2) Gold nanoparticle (AuNPs)/Polyallylamine hydrochloride (PAH) nanocomposite architecture: a self‐assembled AuNPs/PAH achieving relative humidity (RH) range (7‐98 RH%) and 2.3/1.4 s of response/recovery time. 3) AI‐enhanced multimodal decoding: a convolutional neural network (CNN) architecture integrating humidity waveforms with contextual phoneme prediction, achieving 85.61% accuracy in voiceless speech recognition—surpassing EMG‐based systems by 28.71%,^[^ ^39^ ^]^ electroglottographic (EGG) signals by 35.21%, and speech signals by 15.61% in aphonic patients.^[^ ^40^, ^41^ ^]^

## 2 Results

### 2.1 Design of the HSRM System

**Figure 1:**
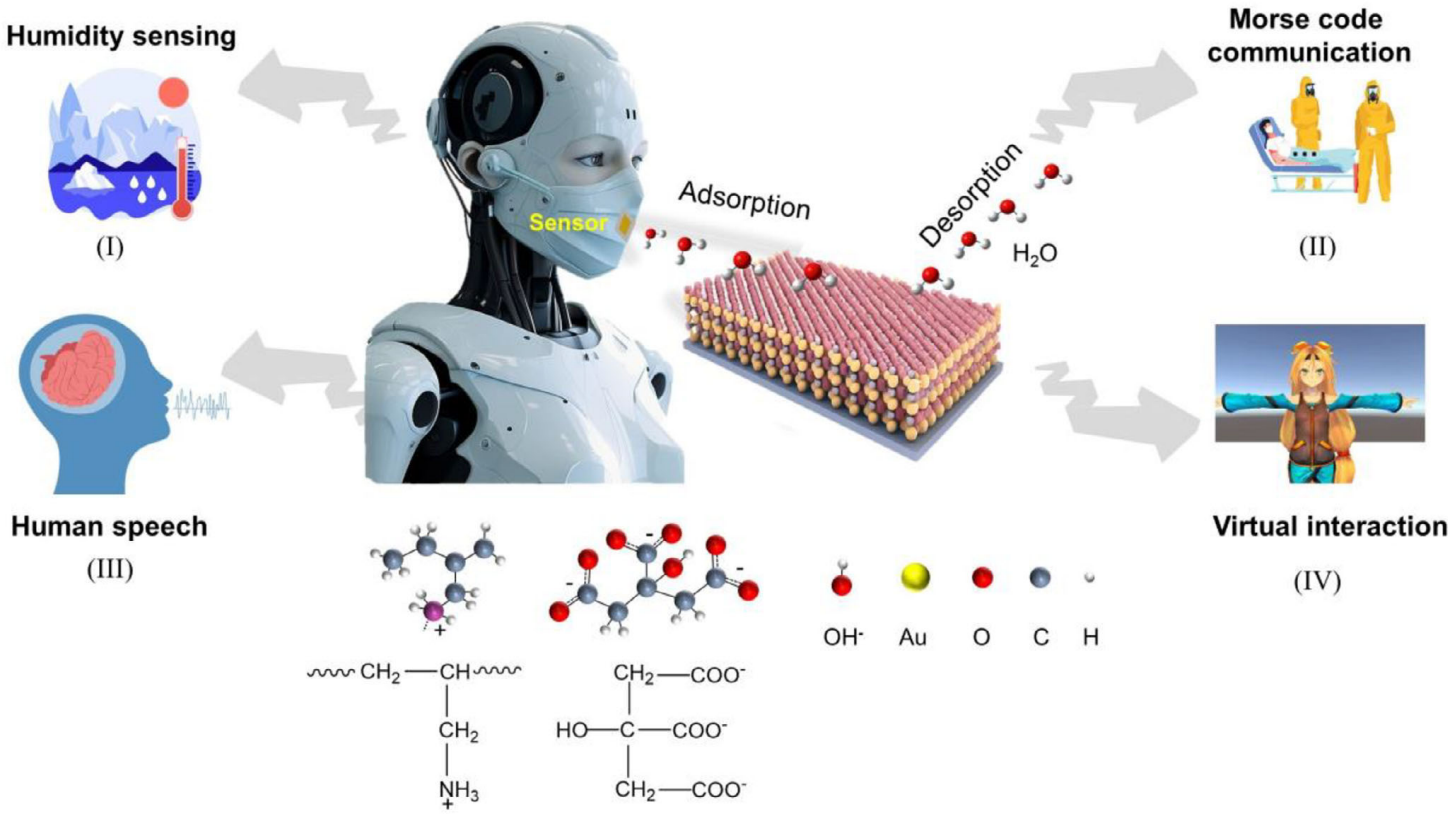
The application schematic of HSRM. I) Sensing humidity changes. II) Communicating by Morse code. III) Speech recognition. IV) Virtual interaction.

The HSRM system is developed utilizing a gold nanoparticle AuNPs/PAH humidity sensor for respiration detection through the self‐assembly of anodic AuNPs and anodic PAH. **Figure** **1** illustrates the operational framework of the device. The heightened sensitivity of the HSRM can be attributed to the substantial surface area ratio of the AuNPs and the presence of numerous water‐absorbing functional groups within the polyelectrolyte PAH. This configuration facilitates the formation of a continuous conductive network of AuNPs, while the distinctive lattice structure of the AuNPs significantly enhances the humidity‐sensing performance of the HSRM. The system exhibits a pronounced swelling effect upon exposure to varying humidity levels. Non‐contact testing experiments were conducted at various distances, including the water surface, fingertip surface, and breath detection, to validate the effective and reliable non‐contact detection capabilities of the HSRM (Figure S1, Supporting Information). The proposed system adopts a plug‐and‐play integration strategy, wherein the sensor (≈1.1 g) is embedded into a commercially available medical mask (≈20 g). This configuration ensures excellent wearability and user comfort, fully aligning with commercial ergonomic standards. Notably, the medical mask retains its built‐in respiratory ventilation pathway, with the detailed structural layout illustrated in Figure S2 (Supporting Information).

### 2.2 Working Mechanism

**Figure 2:**
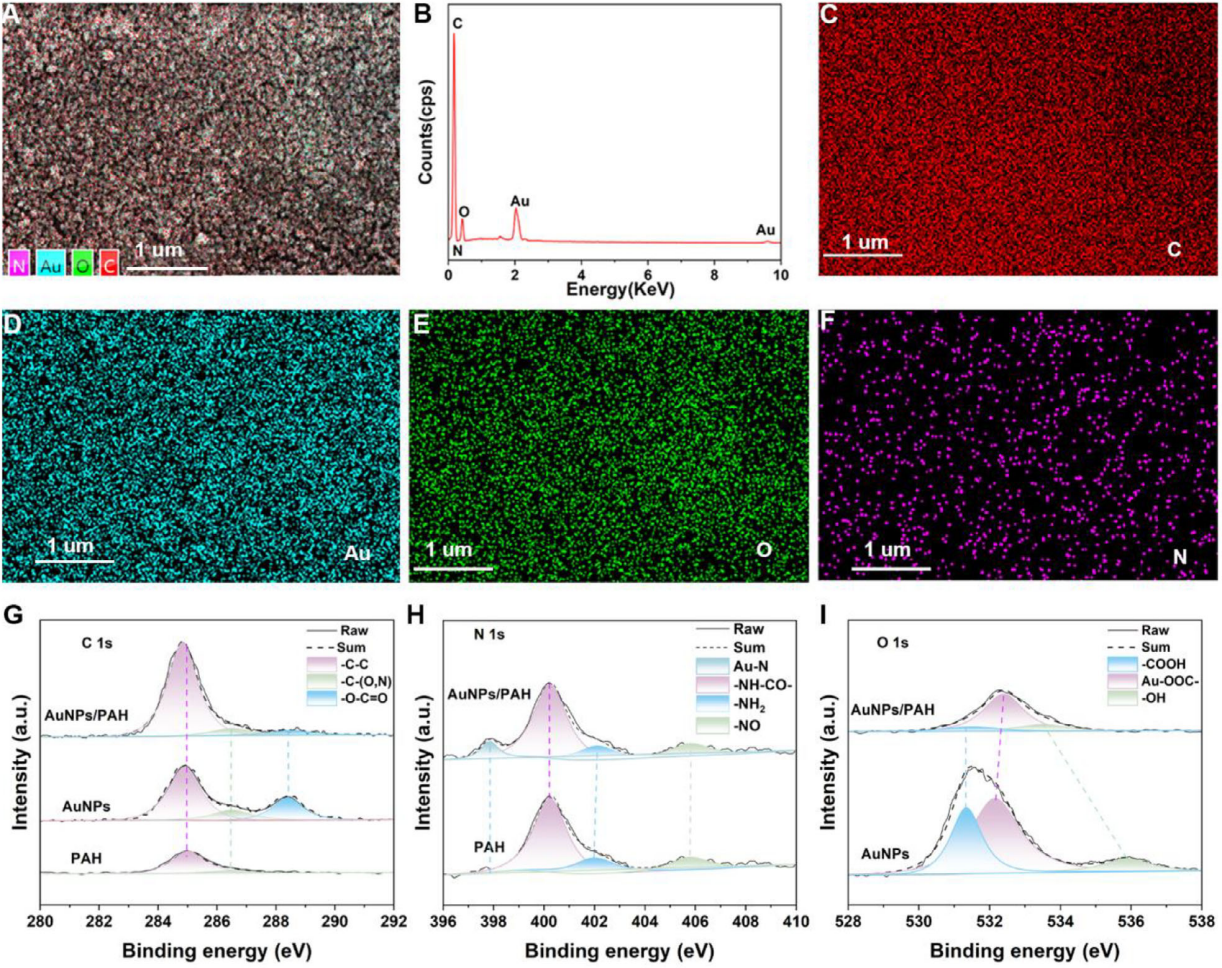
EDS mapping and XPS spectra of the AuNPs/PAH composite. a–f) EDS mapping of AuNPs/PAH. g–i) XPS spectra of deconvoluted C 1s, N 1s, and O 1s in AuNPs, PAH, and AuNPs/PAH.

The influence of humidity on the sensor primarily involves the exchange of water molecules at the interface between the HSRM and the surrounding air.^[^ ^42^ ^]^ Consequently, the specific surface area of the HSRM and the presence of hydrophilic groups within its structure are two critical factors that determine moisture sensitivity.^[^ ^43^ ^]^ The sensing mechanism of the HSRM predominantly pertains to the transfer of electrons from water molecules at the active sites of the sensing material. The elemental distribution of the AuNPs/PAH composite was characterized by Energy‐dispersive X‐ray spectroscopy (EDS) mapping, as shown in **Figure** **2**a–f. Meanwhile, to further investigate the chemical states of the constituent elements, high‐resolution XPS survey spectra were obtained for sodium citrate‐capped AuNPs, pure PAH, and the AuNPs/PAH composite film in Figure S3 (Supporting Information). The magnified views of the N 1s and O 1s XPS spectra are shown in Figure S4 (Supporting Information) for comparison of pure PAH, AuNPs, and AuNPs/PAH composite films, respectively. These spectra confirm the incorporation of N‐ and O‐containing functional groups within the composite structure.

To further elucidate the chemical interactions within the AuNPs/PAH composite films, we investigated the bonding behavior among AuNPs, PAH, and their hybrid structures. The high‐resolution XPS peaks of C 1s, N 1s, and O 1s were deconvoluted using Gaussian fitting models with Origin 2025 and XPSpeak software. The results reveal strong interfacial interactions between AuNPs and the PAH matrix. As shown in Figure 2g, the C 1s peak assigned to ‐C–C undergoes a slight shift from 284.9 eV (in both AuNPs and PAH) to 284.8 eV in the AuNPs/PAH composite, suggesting subtle electronic restructuring. The C–O peak in AuNPs and the C–N peak in PAH, both originally located at 286.5 eV, converge into a unified C–(O,N) signal at 286.5 eV in the composite, indicating the overlap or interaction of O‐ and N‐ containing groups.^[^ ^44^ ^]^ Additionally, the ‐O–C═O signal at 288.4 eV remains consistent in both AuNPs and AuNPs/PAH, further supporting the structural stability of this functional group.^[^ ^45^ ^]^ Figure 2h presents a comparative analysis of the N 1s spectra of PAH and the AuNPs/PAH composite. A distinct peak at 397.85 eV is observed in both samples; however, the AuNPs/PAH spectrum exhibits an additional ligand‐related feature, indicative of a direct interaction between amino groups and the AuNP surface. The primary peak ‐NH–CO– remains at a similar binding energy but displays noticeable broadening in the composite, suggesting an indirect interaction pathway, likely mediated by secondary coordination or hydrogen bonding. Notably, the intensity of the ‐NH₂ signal is significantly enhanced in the AuNPs/PAH film, implying a modulation of surface charge states facilitated by AuNP integration. Furthermore, the ‐NO peak, attributed to oxidative exposure of PAH under ambient conditions, appears as a broadened and flattened feature ≈405.85 eV in the composite. This shift and broadening are consistent with changes in electron density distribution due to complex formation, accompanied by an increase in high‐binding‐energy components likely arising from surface adsorption phenomena or chemical transformations at the interface.^[^ ^46^ ^]^

The peak O 1s spectrum of AuNPs is decomposed into three Gaussian components at 531.35, 532.2, and 535.9 eV in Figure 2i. In the composite, the uncoordinated ‐COOH remains at 531.35 eV, while the coordinated Au–OOC‐ component exhibits a shift toward higher binding energy, indicative of stronger metal–ligand interactions. Meanwhile, the ‐OH component shifts toward lower binding energy, suggesting increased hydrogen bonding or electron delocalization.^[^ ^45^, ^47^ ^]^ Collectively, the observed shifts in C 1s, N 1s, and O 1s binding energies underscore the formation of robust chemical interactions between AuNPs and PAH. These interactions modulate the electron density of polar functional groups—carboxyl, hydroxyl, and amine—enhancing the composite's capacity for water molecule adsorption.

**Figure 3:**
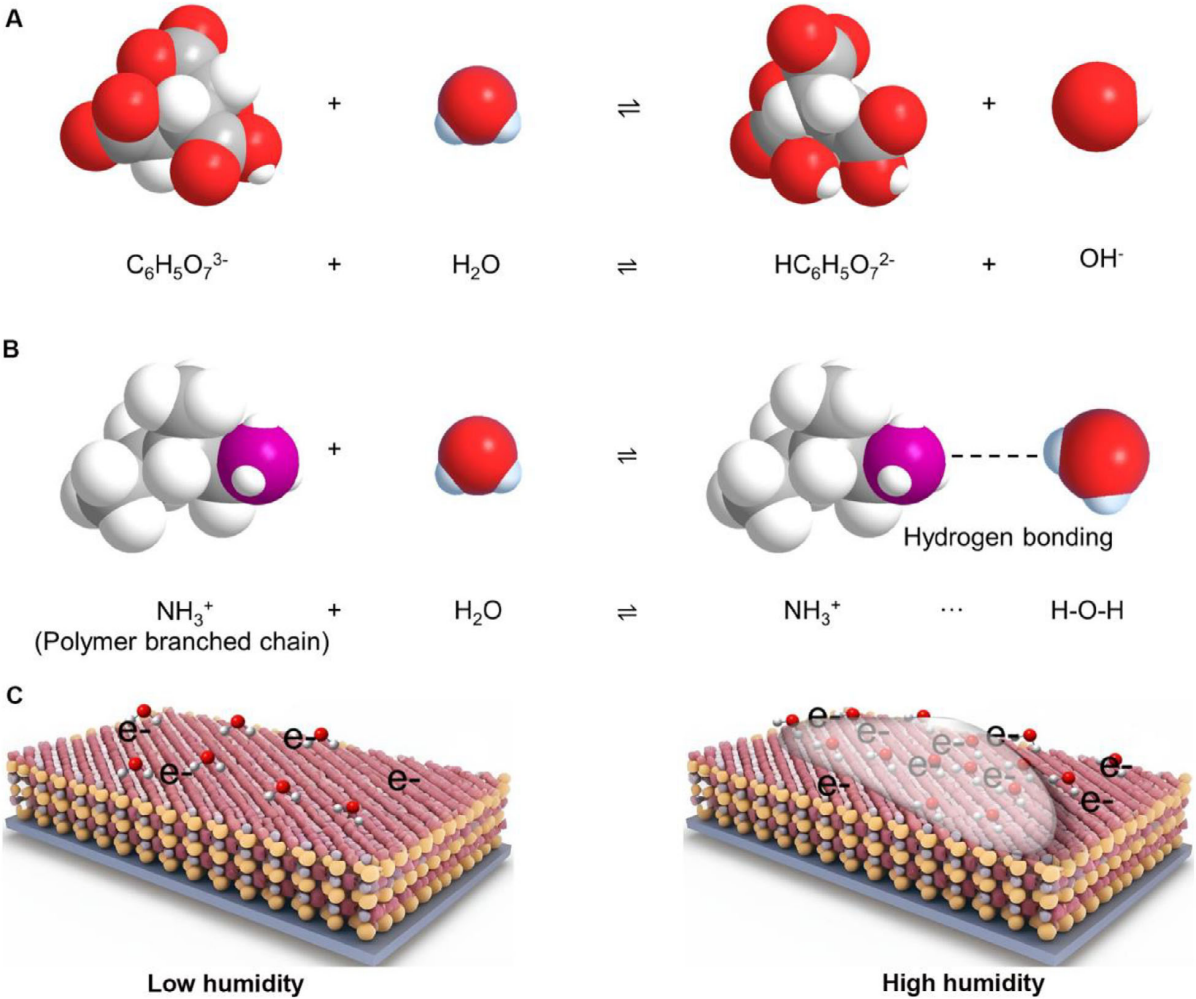
Working mechanism of the HSRM system. a) Equation for the chemisorption of water molecules by C₆H₅O₇^3^⁻. b) Equation for chemisorption of water molecules by NH_3_^+^. c) The working mechanism of HSRM in different RH conditions.

Under low RH conditions, water molecules physically adsorb onto the active sites of the HSRM due to the electropositive nature of PAH and the electronegative characteristics of AuNPs.^[^ ^48^ ^]^ In possessing a significant specific surface area, the surface of AuNPs is coated with sodium citrate, which contains a substantial amount of Citrate (C₆H₅O₇^3^⁻). This coating can expand considerably upon moisture absorption, thereby significantly enhancing the sensitivity of the HSRM to moisture. The relevant chemical reaction is illustrated in **Figure** **3**a. Additionally, PAH, as an amine salt, contains numerous amine groups (NH_3_^+^) within its molecular structure, which readily form hydrogen bonds with water molecules. NH₃⁺ in PAH binds to water molecules through hydrogen bonding in a process that can be expressed in Figure 3b and Figure S5 (Supporting Information) (Equations S1,S2, Supporting Information).

Furthermore, the tunneling effect among donor water molecules increases the free charge on the HSRM. Thus, the primary mechanism at low RH is electron tunneling at the donor water sites. As the RH increases, a greater number of water molecules are absorbed onto the sensor surface, leading to chemisorption. In this phase, water molecules, in addition to the donor water molecules, facilitate proton transfer pathways, thereby enhancing the conductivity of free charge and resulting in a decrease in the sensor's electrical resistance. At this juncture, the predominant sensing mechanism shifts to proton hopping. Consequently, the changes in resistance and the heightened sensitivity of the HSRM can be elucidated through the Grotthuss mechanism.^[^ ^49^ ^]^ At room temperature, superoxide (O_2‐_) plays a pivotal role in the dissociation of water molecules in the reaction illustrated below:^[^ ^50^ ^]^

(1) $$H_{2}O+\frac{1}{2}{O_{2}}^{-}+\frac{3}{2}e^{-}↔2OH^{-}$$

At low humidity conditions, the proton hopping mechanism is inhibited due to physical adsorption processes. The tunneling effect occurs at the active water donor sites, increasing current and decreasing sensor resistance.^[^ ^51^ ^]^ Consequently, the predominant mechanism at low RH is the tunneling effect of electrons at the donor water sites (Figure 3c, left). At higher humidity conditions, the quantity of water molecules adsorbed onto the surface of the active layer increases, leading to chemisorption and the formation of a water film (Figure 3c, right). At this juncture, the predominant conductive mechanism at high RH is proton hopping. Consequently, the primary mechanism at high RH is elucidated by the Grotthuss mechanism. The reaction involving hydrated hydrogen ions that are dissociated from water molecules is presented below^[^ ^52^ ^]^:

(2) $$\begin{array}{c} H^{+}+H_{2}O\to H_{2}O+H^{+} \end{array}$$

In the described reaction, a single water molecule functions as an acid by accepting a hydrogen ion (H^+^) from another water molecule, resulting in the formation of a hydronium ion (H_3_O^+^) and the simultaneous release of a hydroxide ion (OH^−^). Additionally, the H^+^ derived from the dissociation of water is chemisorbed onto the carboxyl site by the citric acid component on the AuNPs, while the hydroxide ion is chemisorbed onto the amino site by PAH, where it serves as an electron donor. Furthermore, water molecules present at the defect sites of the HSRM can also be chemisorbed onto the carboxyl site. Ultimately, the desorption of H_2_O molecules is expedited due to the irregular interstitial structure resulting from the aggregation of AuNPs within the humidity sensor of HSRM, which contributes to the sensor's rapid recovery performance.

### 2.3 Working Performance

**Figure 4:**
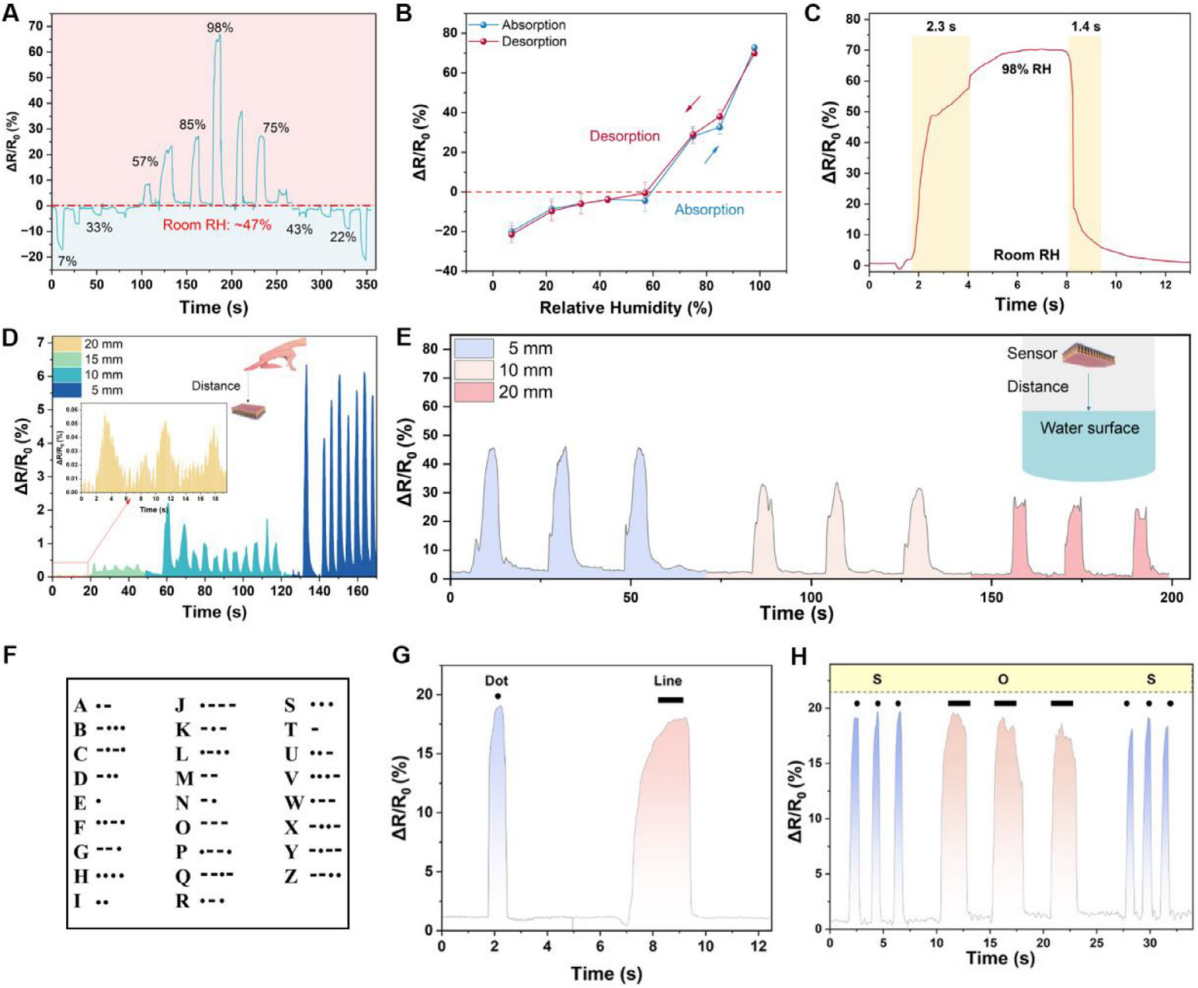
Performance evaluation of the HSRM system. a) Response and recovery curves of the sensor as the RH progressively increases from 7% to 98% and returns at 47% room RH. b) The sensitivity of the HSRM. c) The response/recovery time of the HSRM as the RH is from 47% to 98%. d) Effect of distance between the fingertip and the HSRM on resistance change. e) Effect of distance between the water surface and the HSRM on resistance change. f) Morse code decoding rules. g) Short/shallow means “·”, and long/deep breath means “‐”. h) The signal of “SOS”.

The various salt solutions at saturated concentrations were prepared to create distinct RH environments. The experimental apparatus is illustrated in Figure S6 (Supporting Information). We demonstrated that the contact angle of AuNPs/PAH was 73° (Figure S7, Supporting Information), which suggests our device has good hydrophilicity with adsorption and desorption capabilities. The adsorption and desorption processes of water molecules in the constant humidity beaker are characterized by a gradual increase and then a decrease in RH (7%–98%–7%) at a baseline room RH of 47% (see **Figure** **4**a). We can also notice from the result that when the humidity of the constant humidity beaker is lower than the room RH, the signal of the sensor is a negative change value. When the humidity of the constant humidity beaker is higher than the room RH, the signal of the sensor is a positive change value, indicating the sensitivity of our device to the ambient humidity. The shape and recoverability of the response curves demonstrated the reversibility of the humidity sensor, exhibiting minimal hysteresis. The adsorption‐desorption variation of the sensor with increasing and decreasing RH is shown in Figure 4b. The formula used to calculate sensitivity (S) is as follows^[^ ^53^, ^54^ ^]^:

(3) $$\begin{array}{c} S=\frac{\Delta R/R_{0}}{\Delta RH}\; \end{array}$$

In this study, ΔR represents the change in resistance, R_0_ denotes the initial resistance, and ΔRH indicates the change in humidity. It is important to note that the humidity sensor is inevitably exposed to ambient air during the experiment. Consequently, we conducted further investigations into the adsorption characteristics of humidity sensors when immersed in water, as well as their desorption capabilities upon removal from the water surface. Specifically, we submerged the humidity sensor in water, recorded the signal changes, and subsequently extracted the sensor from the water. The results demonstrate that the sensor is capable of rapidly recovering its initial resistance (Figure S8, Supporting Information). The rapid change in RH from 7% to 98% results in a response time of 2.3 s and a recovery time of 1.4 s for the HSRM, as illustrated in Figure 4c. However, the sensor exhibits varying response times under different humidity conditions. Considering that the RH of human exhaled breath typically ranges from 60% to 90%.^[^ ^55^ ^]^ We evaluated the sensor's response to exhaled gas under 7% RH, 75% RH, and 85% RH humidity environments. As shown in Figure S9 (Supporting Information), both the sensor's response magnitude and recovery time differ significantly with humidity. At 7% RH, the sensor's feedback change is small, but the recovery time is 2.3 s. At 75% RH, the sensor's feedback change value is larger but requires a response recovery time of over 10.3 s. At 85% RH, the sensor's response recovery time is over 22 s. This performance also proves that our device has a wide humidity response range, and provides a research basis and reference direction for developing a customizable high‐precision humidity sensing system.

Furthermore, to assess the sensor's long‐term stability, we employed a custom‐designed human breath simulation system to conduct a 24‐h continuous performance test. During the experiment, the humidity level was maintained at 89% RH using a humidifier, while the contact frequency between the sensor and the humid environment was controlled via a DIY telescopic rod operating at a constant speed of 7 cm s^−1^. All experimental parameters were kept consistent throughout the testing period (see Video S1, Supporting Information). The corresponding results are presented in Figure S10a (Supporting Information). As shown in Figure S10b (Supporting Information), the sensor's performance degradation is limited to only 2% over the 24‐h duration. Additionally, Figure S10c,d (Supporting Information) further confirms the sensor's stable and reliable performance after prolonged operation. And we compare the performance of various types of humidity sensors is provided in Table S1 (Supporting Information).

In addition, the sensing distance of the device was examined to assess the potential application of the HSRM as a non‐contact switch. The sensor was positioned flat, and volunteers were instructed to extend a finger near it. Figure 4d illustrates the variations in signal output as the finger is placed at varying distances from the sensor. Notably, as the distance of the finger decreases from 20 to 5 mm, there is a significant increase in the corresponding sensor response. Furthermore, as the height of the sensor above the water surface is altered from 20 to 5 mm, the sensor's resistance also changes, as depicted in Figures 4e and S11 (Supporting Information). Morse code is an encrypted message transmission method used for emergency communications, and it is an important tool for transmitting information in emergencies.^[^ ^56^, ^57^ ^]^ According to the decoding rules of Morse code in Figure 4f, we represented the signals generated by short/shallow breaths as dots and the signals generated by long/deep breaths as lines, according to the alphabetic representation of Morse code, as shown in Figure 4g. Based on the above rules, the results demonstrate that our sensor can signal “SOS” in special situations using different exhalation patterns (see Figure 4h).

### 2.4 Respiration Language Signals Acquisition

**Figure 5:**
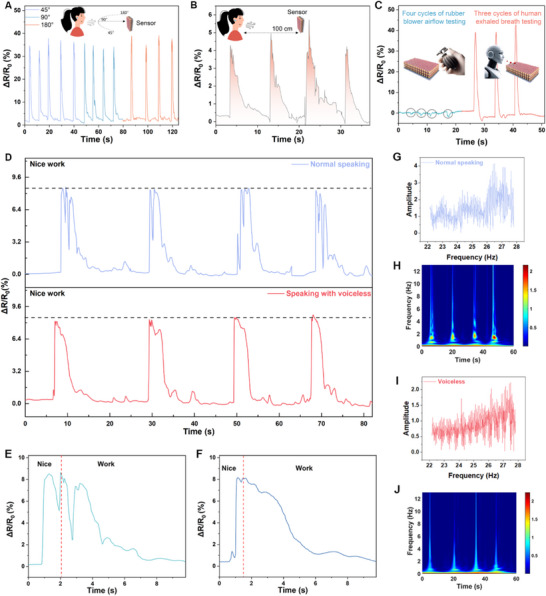
Converting respiration signals into resistance signals. a) Effect of respiratory signals at different angles on resistance changes. b) Change in resistance of respiratory signals detected at 100 mm. c) Comparison of four airflow cycle tests (four black circles) and three human exhaled breath cycle tests. d) Participants pronounced “Nice work” on the HSRM output with vocal fold vibration (upper) and without sound (lower). e) Amplification of a single signal with voice f) Amplified single signal without voice g) Amplitude‐frequency of signals with vocal fold vibrations. h) CWT of signals with vocal fold vibrations. i) Amplitude‐frequency of signals with voiceless. j) CWT of signals with voiceless.

To test the performance of the sensor on exhaled gas, we did further tests from different angles and distances. First, the sensor is capable of detecting breathing signals from multiple angles. As demonstrated in **Figure** **5**a, when a volunteer exhaled toward the sensor from three different directions (45°, 90°, and 180°), the results indicated that the resistance change signal remained consistent regardless of the direction, confirming the device's ability to detect breathing signals within the 0–180° plane. Then, to eliminate the influence of temperature in this experiment, we also subjected the humidity sensor to hot air, which resulted in minimal resistance change (Figure S12, Supporting Information). Additionally, we investigated the resistance of the humidity sensor as the temperature was increased from 30 to 40 °C and subsequently returned to 30 °C, revealing that the range of resistance change was much smaller than that caused by human exhalation (Figure S13, Supporting Information). Figure 5b presents the results of a distance test assessing the sensor's ability to perceive breath signals. In this experiment, the subject stood in front of the distance sensor, maintaining a parallel orientation of the mouth to the sensor, and then began to breathe deeply toward it. The findings indicated that the HSRM could successfully detect breathing signals at a distance of 100 mm.

To test the difference between our device's perception of airflow and human exhaled gas, we used a rubber pump dust blower to blow airflow directly into the sensor four times and recorded the data. We then recorded data from volunteers blowing three times directly into the sensor, and the results are shown in Figure 5c. The results indicate a significant difference between our sensor's airflow sensing and human exhaled breath. The effect of airflow on the sensor is negligible compared to exhaled breath. After obtaining the test results regarding the performance and initial application of the HSRM, we directed our attention toward utilizing the HSRM for the collection of wheezed speech signals, specifically, the articulation of phrases without the production of audible sound, as shown in Figure S14 (Supporting Information) It was necessary to eliminate the interference caused by acoustic signals generated during speech. We compared five consecutive signals of normal and voiceless speech to assess the accuracy of the purely breathy signals in conveying speech, as depicted in Figure 5d. In this instance, the participant was instructed to articulate the phrase “Nice work” in both the voiced condition (with vocal folds vibrating) and the unvoiced condition (without vocal fold vibration). Figure 5e,f illustrates the differences in signals between the participant's vocal folds vibrating and not vibrating while articulating “Nice work.” The signal is more pronounced when vocal vibration occurs. Figure 5g,h presents a comparison of the amplitude‐frequency plots and continuous wavelet transform plots corresponding to vocal fold vibration, respectively. Figure 5i,j provides a comparison of the amplitude‐frequency plots and continuous wavelet transform plots for vibrations occurring without sound. Notably, the amplitude of the voiced signal was significantly greater than that of the voiceless signal at low‐frequency ranges.

### 2.5 Controls Virtual Character Speech by Respiration Language

**Figure 6:**
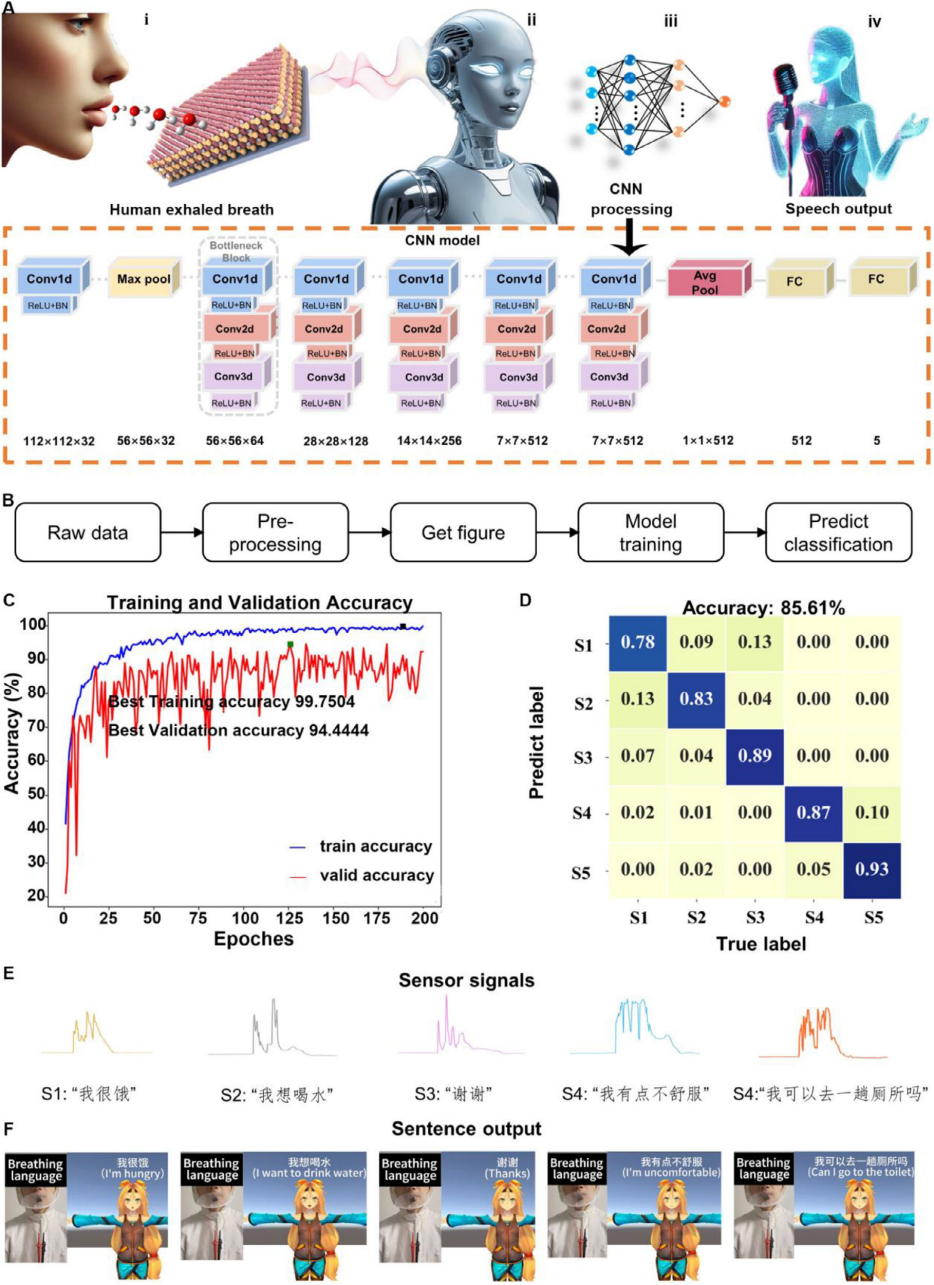
Demonstration of CNN‐assisted breath language detection for recognizing modality. a) CNN modules used in respiratory language systems. b) Flowchart of the data processing process. c) The best accuracy plots of training (black dot) and validation data (green dot). d) Confusion matrix of five volunteers with an overall accuracy of 85.61%. e) The sensor captures the exhaled breath signal when the volunteer only makes the speaking motion. f) Virtual character voice output that corresponds to the breathing signals of the user making speaking movements.

The semantic analysis of the signal was conducted using classification learning techniques. Utilizing respiration‐generated data, a CNN algorithm was employed to classify and recognize various respiration signals, subsequently matching them with the corresponding speech signals for output through the system components. This application is particularly relevant for individuals who are temporarily unable to speak due to laryngeal surgeries. To illustrate the training algorithm, five commonly used Chinese sentences were selected: S1: “我很饿” (I'm hungry), S2: “我想喝水” (I want to drink water), S3: “谢谢” (Thanks), S4: “我有点不舒服” (I'm uncomfortable), and S5: “我可以去一趟厕所吗？” (Can I go to the toilet?). Five healthy male volunteers aged 20–30 years were recruited to complete the test. Given that breath signals can be accurately detected and recognized through electrical signals, we developed a comprehensive schematic for the collection of silent speech data, transitioning from mouth breathing to speech output, as illustrated in **Figure** **6**a.

The process involves the following steps: i) Data collection: Participants speak silently into the sensor without vocal, allowing the exhaled breath produced during speech to activate the sensor. ii) Data processing: After basic processing, which includes the removal of outliers, the collected data is segmented to create data images for each experiment. iii) Model architecture: The collected data is processed using a CNN with a 50‐layer architecture. It begins with an initial Conv2d layer (64 filters, kernel size: 7 × 7, stride: 2) followed by a max pooling (Maxpool2d) layer for down‐sampling. The network is organized into four bottleneck stages, each containing multiple Conv2d layers with 64, 128, 256, and 512 filter sizes. Each layer is paired with batch normalization (BN) and ReLU activation, while shortcut connections in the bottleneck blocks improve gradient flow and efficiency. Pooling operations, including Maxpool2d and average pooling (Avgpool), reduce spatial dimensions and aggregate features. The final stage includes an Avgpool layer, followed by a fully‐connected (Fc) layer with a softmax activation function, which outputs the classification results. The detailed layer structure and parameters are shown in Figure S15 (Supporting Information). iv) Virtual speech output: The processed data is then used to generate virtual character speech output, which mimics the user's intended speech action.

To demonstrate the capability of the humidity sensor in detecting different spoken sentences, a CNN model with five output classes, corresponding to five example sentences, was utilized. Experimental data on humidity changes during the pronunciation of these sentences was collected to train the model. The overall process of classification learning is illustrated in Figure 6b. Initially, data were collected from five participants using the sensor. To ensure data quality, samples exhibiting significant errors—such as inaccuracies in data collection or instances of missing data—were removed. The remaining data were converted into graphs, which served as input for the CNN model. The dataset consisted of 2489 samples, with 80% randomly selected as training data and 3% for the validation data, and 17% for the test data. Each sample included the temporal voltage signal captured by the sensor, which was preprocessed and fed into the CNN. The input data was represented as images, capturing the dynamic humidity variations over time during speech. Given the nonlinear and dynamic characteristics of the voltage signal, the CNN architecture was designed to progressively extract spatial and temporal features from these input images.

Specifically, the network utilized five layers of residual Bottleneck blocks, with ReLU activation functions applied between layers to capture the complex dependencies in the data. After feature extraction, the output was passed through a fully connected layer and mapped to five output classes. The cross‐entropy loss function was employed to measure the classification error, and the gradient descent method was used to optimize the model parameters. During training, the network was observed to converge after ≈25 epochs, achieving an accuracy of ≈90% on the test set. The model was then trained to convergence, as shown in Figure 6c, using the training and validation sets. The results indicated that the highest training prediction accuracy achieved during the model training was 99.75% (marked by the black dot on the blue line), while the highest accuracy for the validation set was 94.44% (indicated by the green dot on the red line). After training, the test set was input into the trained model to generate prediction results. The model's performance on the test set is summarized in the confusion matrix (see Figure 6d). The model achieved an overall prediction accuracy of 85.61%, demonstrating its ability to generalize across different participants and effectively predict respiratory patterns.

Finally, the pre‐recorded speech signals are generated using the respiratory system component, as shown in Figure 6e, which displays the voltage signals corresponding to the respiratory language. The processed signals are integrated with Unity software, enabling virtual control technology that matches the breathing signal with the virtual character's voice actions. The resulting output is shown in Figure 6f (Video S2, Supporting Information). Users only need to open their mouths and perform speech motions for the virtual character to automatically recognize their breathing signals and generate the corresponding speech output. This system has the potential to significantly enhance communication efficiency for patients recovering from laryngeal surgery.

## 3 Discussion

Through the integration of artificial intelligence (AI) and flexible electronic skin technology, the non‐contact humidity‐sensing respiratory language system promises to usher in a new era of human‐computer interaction (HCI). Extensive research efforts, such as Google's Project Euphonia and various commercially available electronic larynx devices,^[^ ^58^ ^]^ have explored alternative speech technologies that do not rely on vocal fold activity. Specifically, Project Euphonia utilizes advanced AI techniques to significantly improve speech recognition capabilities for individuals with speech impairments, such as amyotrophic lateral sclerosis (ALS),^[^ ^59^ ^]^ cerebral palsy, and post‐stroke speech disorders,^[^ ^60^, ^61^ ^]^ positioning it among the most impactful disability‐focused speech recognition endeavors. Nevertheless, challenges remain regarding the limited availability of training data and the need for further improvements in real‐time optimization. In addition, in the commercial electronic larynx devices application cases we investigated, the main target group is patients after laryngeal surgery,^[^ ^62^ ^]^ who sometimes have swollen necks and can't produce neck vibration, resulting in the inability to realize voice communication (Table S2, Supporting Information).^[^ ^63^, ^64^ ^]^

Our respiratory humidity sensing microphone system avoids these problems. The plug‐and‐play integration is easy to wear and use, and we are currently focusing on providing a generalized database of daily life conversations to help patients better recover from surgery. It can also be integrated into a ventilator to allow patients to engage in simple speech through breathing. In our current tests, our main research goal is to test breathing under normal breathing conditions in healthy users aged 20–30 years old. The HSRM system employs a wired data acquisition configuration, utilizing a Keithley 7510 digital multimeter for all signal measurements. While this setup ensures high measurement accuracy under controlled laboratory conditions, it presents notable limitations in terms of portability and operational flexibility. Specifically, the reliance on wired connections and external power supply restricts mobility and continuous use, thereby falling short of the practical requirements for real‐time, on‐the‐go monitoring in clinical or wearable healthcare applications.

As a next step, we will further develop the mini‐device and expand the respiratory language database to include people of different ages, health conditions, and language styles for testing, to improve the versatility of the device and provide patients with a better tool for laryngeal rehabilitation. We will focus on optimizing miniaturization and portability to achieve breakthroughs in device performance through systematic technical innovation. This research plan outlines the phased development of core technologies. First, the hardware architecture will be reconfigured by adopting a microcircuit module integration scheme, eliminating traditional cable connections via flexible printed circuit technology. Second, an intelligent data acquisition platform will be developed to enable dual‐mode operation, encompassing both data preprocessing and real‐time transmission. At the algorithmic layer, a lightweight machine learning model will be introduced to simultaneously improve data processing efficiency and the accuracy of sensor feature recognition.^[^ ^65^ ^]^ The proposed energy supply system will employ a hybrid storage scheme, comprising micro‐supercapacitor arrays integrated with innovative humidity gradient‐based energy collection devices to facilitate continuous power capture from environmental humidity fluctuations.^[^ ^66^, ^67^ ^]^ Through deep integration of AI and flexible electronic skin, a non‐contact humidity‐sensing respiratory language system can open a new era in HCI. This system captures subtle humidity fluctuations in respiratory airflow and employs an adaptive neural network to analyze linguistic features. As a result, it could eliminate reliance on vocal cord vibration or skin contact, enabling a truly contactless communication method.

## 4 Conclusion

Non‐vocal‐triggered speech systems represent an innovative trend in the development of future speech microphones, particularly in healthcare. These systems hold significant potential for enabling individuals, such as singers, teachers, and other professionals with damaged laryngeal vocal cords, to regain their ability to communicate during the recovery process. In this study, we demonstrated an intelligent HSRM system by strategically integrating citrate‐functionalized AuNPs/PAH nanocomposites into a conformal facial interface. The sensing mechanism exploits the synergistic moisture‐capturing capabilities of citrate‐stabilized AuNPs and the humidity‐responsive ammonium groups in PAH, collectively enabling dynamic modulation of ionic conduction pathways through solvation‐induced structural reorganization. This unique nanomaterial interaction produces quantifiable electrical signal variations proportional to respiratory humidity fluctuations. Experimental validation confirms the system's dual functionality in both contact‐mode respiration analysis and non‐contact interface applications. The engineered sensor exhibits rapid humidity response characteristics suitable for real‐time breath pattern recognition, coupled with consistent non‐contact detection capabilities across multiple operational distances. Notably, by transcending traditional limitations of surgically implanted or skin‐adhered devices, our device established a new frontier in assistive communication technology. Besides, by converting respiratory humidity patterns into linguistic signals through the nanomaterial‐engineered sensing device and deep learning interpretation, the HSRM system empowers vocal‐impaired individuals to regain naturalistic communication capabilities without surgical implants or skin‐contact hardware.

Looking ahead, by enhancing the environmental robustness of the nano‐sensing interface and the contextual understanding capabilities of the deep learning model, the system has the potential to integrate with brain‐computer interfaces for direct thought‐to‐speech conversion by enhancing the environmental robustness of the nanosensing interface and the contextual understanding of the deep learning model. The non‐invasive and highly covert nature of this approach has the potential to redefine the design paradigm of assistive communication devices, providing a more natural and seamless mode of expression for voiceless individuals and inspiring a new generation of wearable devices. Ultimately, this technological breakthrough represents a transition from “replacing missing functions” to “reconstructing the essence of communication,” laying the foundation for a truly barrier‐free society.

## 5 Experimental Section

### Materials and Equipment

Chloroauric acid (HAuCl_4_), Sodium citrate, and Poly (allylamine hydrochloride) (PAH, molecular weight = 17 500) were procured from Sigma–Aldrich. All chemicals were utilized without any additional processing. Ultrapure water, with a resistivity of 18.2 MΩ·cm, was employed in all experimental procedures. The polyester film used was high‐density polyester (PET, Young's Modulus 300 MPa), manufactured by DuPont, with a thickness of 200 µm. Characterization was conducted using XPS, EDS, and Helios G4 CX field emission high‐resolution focused ion beam (FIB). The homemade test system was purchased from Taobao (Shengda Machinery Co.). The output signal was recorded using a Keithley 7510 digital multimeter.

### Synthesis of AuNPs

Preparation of AuNPs based on previous and existing studies.^[^ ^68^ ^]^ AuNPs were prepared using the standard reduction method involving sodium citrate. Specifically, aqueous sodium citrate solution (2 mL, 1% w/v) was introduced to a boiling solution of HAuCl_4_ (100 mL, 1 mm). The resultant mixture was subjected to magnetic stirring and heating for 10 min. Subsequently, the heat source was removed, and stirring was continued until the mixture reached ambient temperature. The final solution exhibited a wine‐red coloration, with an average nanoparticle diameter of ≈18±3 nm. Following synthesis, sodium citrate effectively coated the surface of the AuNPs, serving as a stabilizing agent. Under standard pH conditions, these AuNPs displayed a moderate negative charge.

### Fabrication of HPHS

Fabrication of the AuNPs/PAH film concerning previous and existing studies.^[^ ^69^ ^]^ Initially, a suitably sized polyester film was selected to serve as the flexible substrate. Subsequently, the PET was treated with fresh Piranha solution (3:1 (v/v) H_2_SO_4_:H_2_O_2_) for 30 min, followed by thorough rinsing with an ample volume of ultrapure water to ensure complete drying. The AuNPs/PAH film was fabricated using the layer‐by‐layer self‐assembly (LBL‐SA) deposition method. Given that the AuNP was synthesized with sodium citrate, it possessed a negatively charged surface due to the presence of sodium citrate. The process commenced with the deposition of a layer of positively charged PAH onto the PET substrate to facilitate the adhesion of the AuNP. Specifically, the substrate was immersed in a 0.1% PAH solution for 10 min, after which it was rinsed with ultrapure water and dried. Following this, the PET was immersed in the AuNP solution for 12 h, followed by two rinses with ultrapure water. This sequence was repeated until the desired number of membrane layers was attained.

### Data Processing

The datasets utilized in this study were compiled from five participants, each of whom engaged in tests involving five Chinese sentences (S1: “我很饿”, S2: “我想喝水”, S3: “谢谢”, S4: “我有点不舒服”, S5: “我可以去一趟厕所吗”). Five volunteers were receuited to evaluate five sentences, collecting 100 experimental data points for each sentence, resulting in a total of 2500 data points. During data processing, erroneous entries, such as missing values, were removed. After cleaning, the final dataset comprised 2489 valid samples. The data was partitioned such that 80% was designated as the training set, 3% as the validation set, and the remaining 17% constituted the test set. After basic processing, which includes the removal of outliers, the collected data is segmented to create data images for each experiment. The output feature map can be calculated according to Equation (4):

(4) $$\begin{array}{c} Output\;\left(i,j\right)=\sum_{m=0}^{K-1}\sum_{n=0}^{K-1}Input\left(i+m,j+n\right)\times Kernel\left(m,n\right)\; \end{array}$$

where K is the size of the convolution Kernel, (*i*, *j*) represents the position of the output feature map, and (*m*, *n*) are the indices of the convolution Kernel.

The CNN model employed in this study primarily comprises convolutional layers, a pooling layer, and a fully connected layer. Initially, the image data underwent pre‐processing to standardize the size specifications and grayscale value ranges of all input images, thereby ensuring consistency in the data format. Subsequently, the normalized image data was fed into a deep‐learning model for training purposes. This research utilized a 50‐layer CNN as the training model. Throughout 200 iterative training rounds, the network parameters were continuously optimized until the model converged, and the training process was completed. Finally, the test data was input into the trained model to perform a predictive analysis of semantic information.

### HSRM and Speech Interaction in Virtual Spaces

For the HSRM interaction system, a virtual character in the Unity software and a linguistically recorded speech signal were used. First, the breath data from the speech was collected and the results were output by a trained model and subsequently imported into the computer. The predictive model was a previously trained CNN classifier and the data used for model training was obtained from the subjects. During the HSRM interaction, the data containing a complete exhaled language needs to be matched with a pre‐recorded speech signal. The predicted commands were then sent to Unity to control the virtual character to emit the corresponding speech.

### Statistical analysis

All data shown in this manuscript are representative. The data quantities in Figure 3b are given as mean and standard deviation. Data processing methods are provided in the subsection “Data processing”. Statistical analyses were performed using Origin, MATLAB, and Python.

## Conflict of Interest

The authors declare no conflict of interest.

## Author Contributions

J.W. and H.Z. have contributed equally to this work. W.J.L. and Z.W. supervised and guided the project. W.J.L. and J.W. conceived and designed the project. J.W, M.G, H.W., and Z.Z. collected and analyzed the data. H.Z. provided CNN data processing and virtual interaction techniques. X.W. provided data image color‐matching guidance. K.M. and W.J.L. provided guidance on the gold nanoparticle fabrication process. J.W. and H.Z. drafted the manuscript. K.M., W.J.L., and Z.W. critically revised the article.

## Supporting information



Supporting Information

Supplemental Video 1

Supplemental Video 2

## Data Availability

The data that support the findings of this study are available on request from the corresponding author. The data are not publicly available due to privacy or ethical restrictions.
