# Supplementary material for: A Wearable AI‐Driven Mask with Humidity‐Sensing Respiratory Microphone for Non‐Vocal Communication
Source: Adv Sci (Weinh). 2025 Jun 27;12(33):e04343. doi: 10.1002/advs.202504343 (PMC12412612; doi:10.1002/advs.202504343)
Supplement: Supplementary file 1 — Supporting Information [file ADVS-12-e04343-s001.docx]

**Supporting information**

**A Wearable AI-Driven Mask with Humidity-Sensing Respiratory Microphone for Non-Vocal Communication**

Jianfei WANG^1, ‡^, Hongyu ZHANG^2, ‡^, Xiaomin WU^3^, Mingyan GAO^4^, He WEN^1, 4^, Zhibo ZHANG^1^, Kremena MAKASHEVA^5^, Wen Jung LI^2, *^ and Zuobin WANG^1, 4, *^

^1^ International Research Centre for Nano *Handling* and Manufacturing of China, Changchun University of Science and Technology, Changchun, Jilin, 130022, China

^2^ CAS-CityU Joint Laboratory for Robotic Research, Department of Mechanical Engineering, City University of Hong Kong, Kowloon, Hong Kong, China

^3^ College of Medical Informatics, Chongqing Medical University, Chongqing, 400016, China

^4^ Centre for Opto/Bio-Nano Measurement and Manufacturing, Zhongshan Institute of

Changchun University of Science and Technology, Zhongshan, 528437, China

^5^ Laboratory on Plasma and Conversion of Energy (LAPLACE), CNRS, Toulouse, France

^‡^ These authors have contributed equally to this work

^*^Co-contact authors: [wenjli@cityu.edu.hk](mailto:wenjli@cityu.edu.hk) and [wangz@cust.edu.cn](mailto:wangz@cust.edu.cn).

**Table S1**. Comparison of the sensing performance of the resistance humidity sensors.

| **Sensing material** | **Detection range (RH%)** | **Response/recovery time (s)** | **Max.**  **Sensitivity** | **Ref.** |
| --- | --- | --- | --- | --- |
| CNCs | 4-95 | 1.9/1.5  0-16% RH | 12.2/80  ΔR/R_0_(%)/(%RH) | ^[1]^ |
| GO | 11-97 | 2/35  (11% RH - 97% RH) | 1.113  (Ω/Ω-RH%) | ^[2]^ |
| MXene/PAAS | 7-80 | 2.2/1.05  (7%RH - 70%RH) | 6.46 (ΔR/R_0_)/ (g/m^2^/h) | ^[3]^ |
| NaCl-modified paper | 5.6-90 | 1208/537  (15% RH - 40% RH) | - | ^[4]^ |
| 2-SA/ PAN | 0-97 | 14.5/2  (0 - 97% RH) | 63.33 Hz/%RH | ^[5]^ |
| h-BN flakes | 5-100 | 10^-4^/10^-4^  (Breathing model) | 2×10^6^ (ΔR/R) or  1.8×10^10^ Ω/%RH | ^[6]^ |
| AMHS | 20-85 | 0.47 / 0.81  (45% RH - 85% RH) | 66800% (ΔI/I_0_) | ^[7]^ |
| MXene/TPU | 11-94 | 16/32  (84% RH) | - | ^[8]^ |
| Carbon Ink/Filter Paper | 11-98 | 132/50  (11% RH - 98% RH) | - | ^[9]^ |
| AMP | 10-95 | 0.9/0.9  (45% RH - 95% RH) | 704 (R_low_/R_high_) | ^[10]^ |
| h-BN | 11-85 | 3/5.5  (11% RH - 85% RH) | 28384% (ΔI/I_dry_) | ^[11]^ |
| ZrSe_2_ flakes | 15-80 | 1/2  (15% RH - 80% RH) | 68 kΩ /%RH or  10^3^ (ΔR/R) | ^[12]^ |
| MoS_2_ flakes | 10-95 | 8/22  (85% RH - 87% RH) | 530  ((I_RH_-I_10_)/I_10_) % | ^[13]^ |
| EBFG | 50-92.4 | 0.092/0.1  (Breathing model) | - | ^[14]^ |
| Gold nanoparticles | 7-98 | 2.3/1.4  (47% RH - 98% RH) | 70/51  ΔR/R_0_(%)/(%RH) | **This work** |

CNCs: Carbon nanocoils; GO: graphene oxide; PAAS: olyacrylate sodium; SA/PAN: sodium alginate/polyacrylonitrile; h-BN: hexagonal boron nitride; AMHS: anodic aluminum oxide assisted MoS_2_ honeycomb structure; AMP: alkalized MXenes/polydopamine; MXene/TPU: MXene/thermoplastic polyurethane; ZrSe_2_: Zirconium diselenide; MoS_2_: Molybdenum disulphide; EBFG: eccentric fiber Bragg grating.

**Table S2**. Comparative analysis of different types of speech devices

| **-** | **References** | **Evaluation Indicators** | **Principles** | **Limitations** |
| --- | --- | --- | --- | --- |
| Google Project Euphonia | Tobin et al^[15]^. | 33% improvement | Fine-tuning a large ASR model (USM) with<1% disordered speech data (~1 000 hours). | Data diversity and computational cost issues. |
|  | Anushiya Rachel et al^[16]^. | Normal EEG： 98.28%  Pathological EEG: 96.90% | Phase-difference analysis of differenced EGG signals. | Dependent on EGG devices, complex calculations and insufficient real-time performance. |
|  | Shor et al^[17]^. | Word error rate: 62% improvement | Fine-tuning models with limited speaker-specific data. | High cost of personalized storage. |
| Commercial Electrolarynx | Rose et al^[18]^. | Mean Intelligibility: 45% | Neck vibration with matching mouth pattern. | Vulnerable to environmental noise, handheld, requires extensive training. |
|  | Sato et al^[19]^. | Satisfaction：60% | Neck vibration with matching mouth pattern. | Vulnerable to environmental noise, handheld, requires extensive training. |
|  | Tuinman et al^[20]^. | Effective: 40% | Neck vibration with matching mouth pattern. | Vulnerable to environmental noise, handheld, requires extensive training. |
| Wearable  Sensor | **Our work** | Accuracy 85.61% | Non-contact humidity sensing, independent of the environment, plug and play, and personalization accessibility. | Need to develop miniaturized wireless sensing devices and customized databases. |


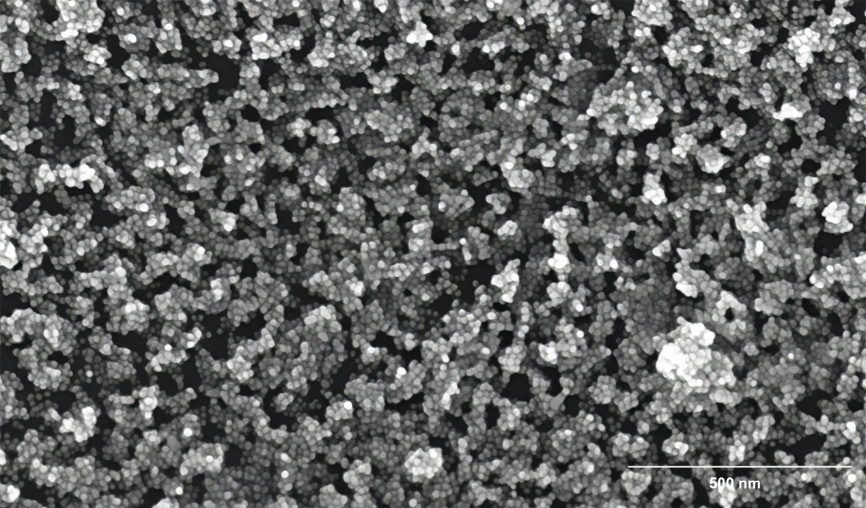


**Figure S1**. Characteristics of the humidity sensor


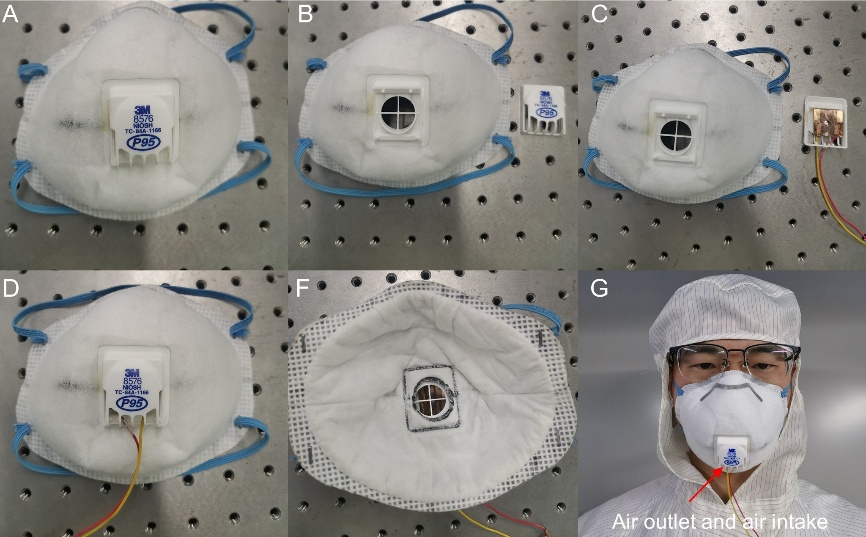


**Figure S2**. The layout of the humidity sensor in HSRM


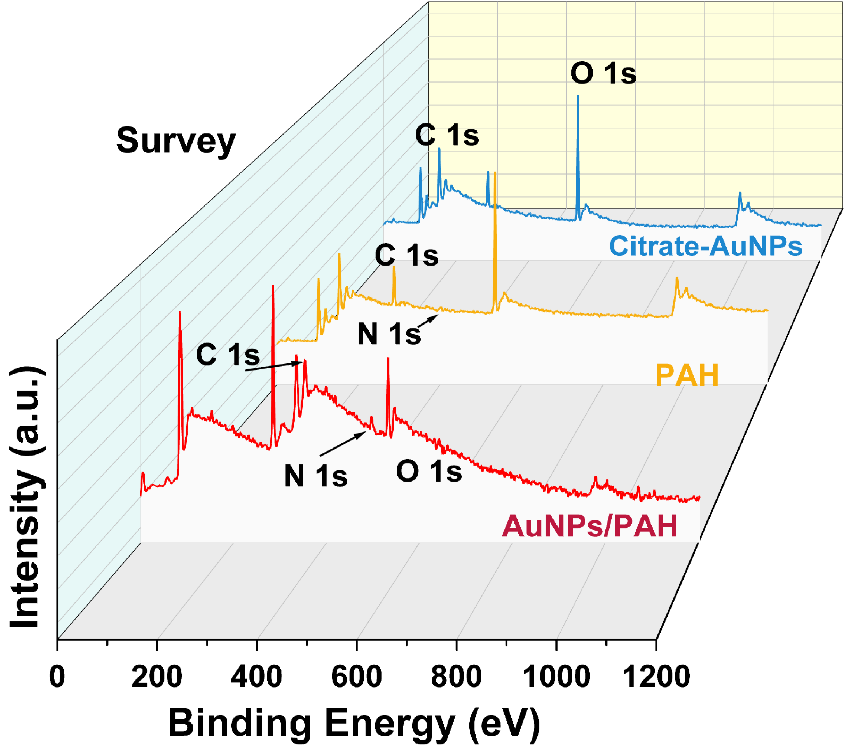


**Figure S3**. XPS survey spectra of AuNPs, pure PAH, and the AuNPs/PAH composite film


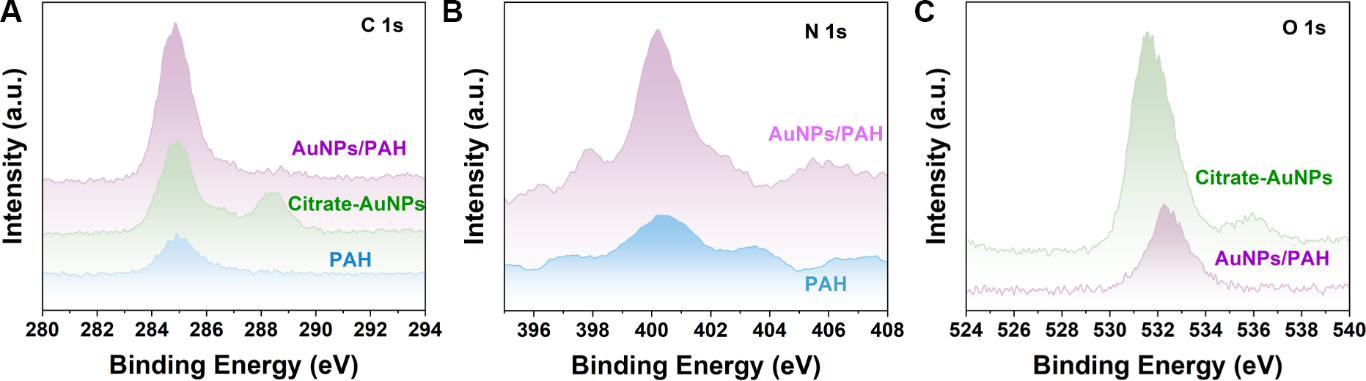


**Figure S4**. The magnified views of the N 1s and O 1s XPS spectra of AuNPs, pure PAH, and the AuNPs/PAH composite film

Citrate (C₆H₅O₇³-) is a conjugate base of a weak acid (citric acid) and will partially hydrolyse to produce a small amount of OH^-^, making the solution weakly basic. Chemical equation as following:

$$\begin{aligned} C_{6}H_{5}{O_{7}}^{3-}(aq)+H_{2}O(l)\leftrightarrow{HC}_{6}H_{5}{O_{7}}^{2-}(aq)+OH^{-}(aq)\#\left( S1 \right) \end{aligned}$$

In the situation of physical hydration: C₆H₅O₇³^-^ Binds to water molecules by ion-dipole interaction to form hydrated ions. There is no breaking or formation of chemical bonds involved here, only adsorption of water molecules by electrostatic action.

In the situation of chemical hydrolysis: A weak hydrolysis reaction of citrate with water molecules, producing a small amount of OH^-^, is a process of chemical equilibrium.

NH₃⁺ in poly(allylamine hydrochloride) binds to water molecules through hydrogen bonding in a process that can be expressed as:

$$\begin{aligned} N{H_{3}}^{+}(polymer branched chain)+H_{2}O\leftrightarrow N{H_{3}}^{+}\cdots H-O-H\#\left( S2 \right) \end{aligned}$$

Oxygen atoms (partially negatively charged) in a water molecule combine with hydrogen atoms (partially positively charged) in NH₃⁺ by electrostatic forces. The schematic of polymer fragment is shown in **Figure S5**.


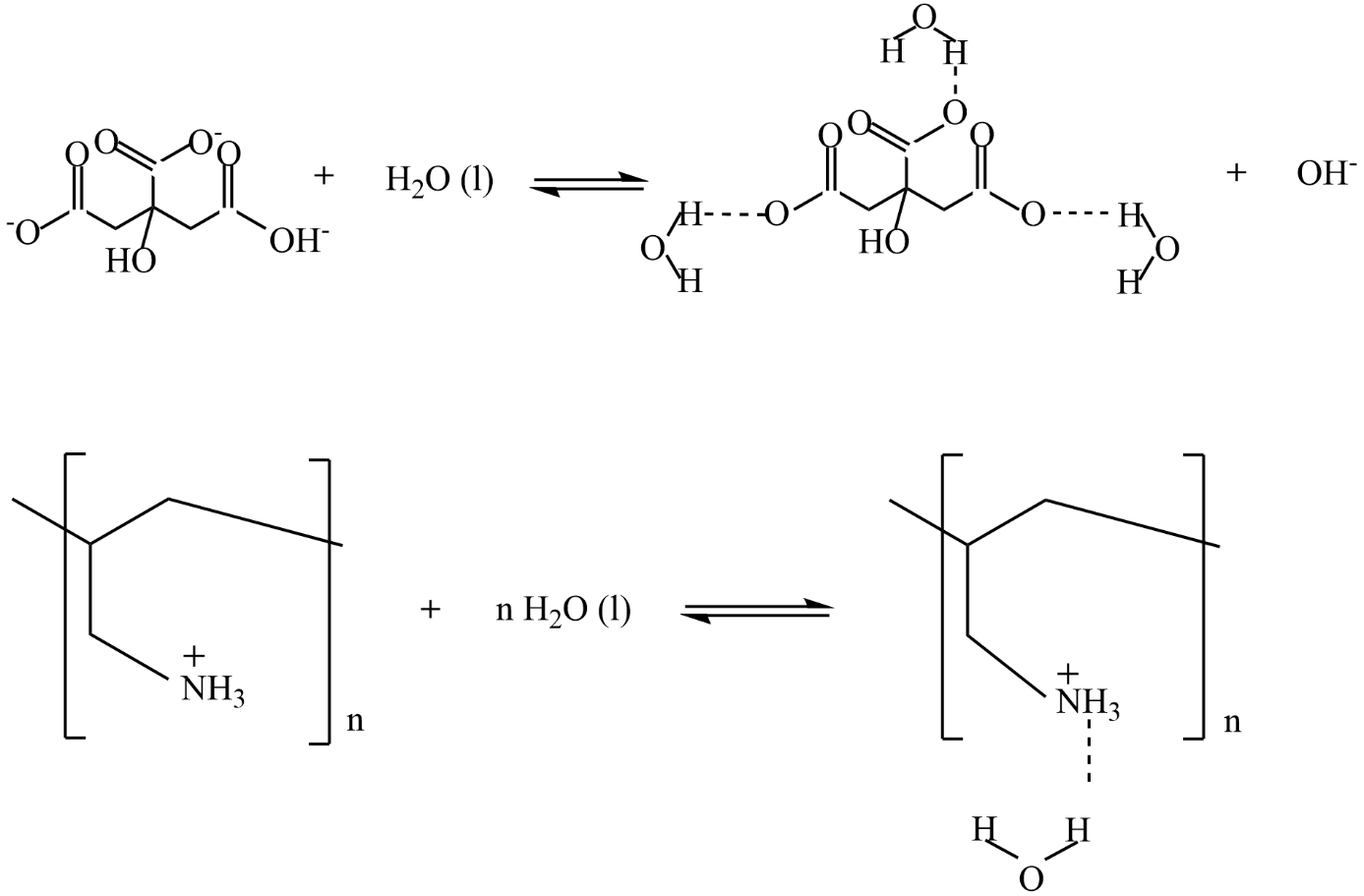


**Figure S5.** Schematic diagram of polymer fragments bound to water molecules


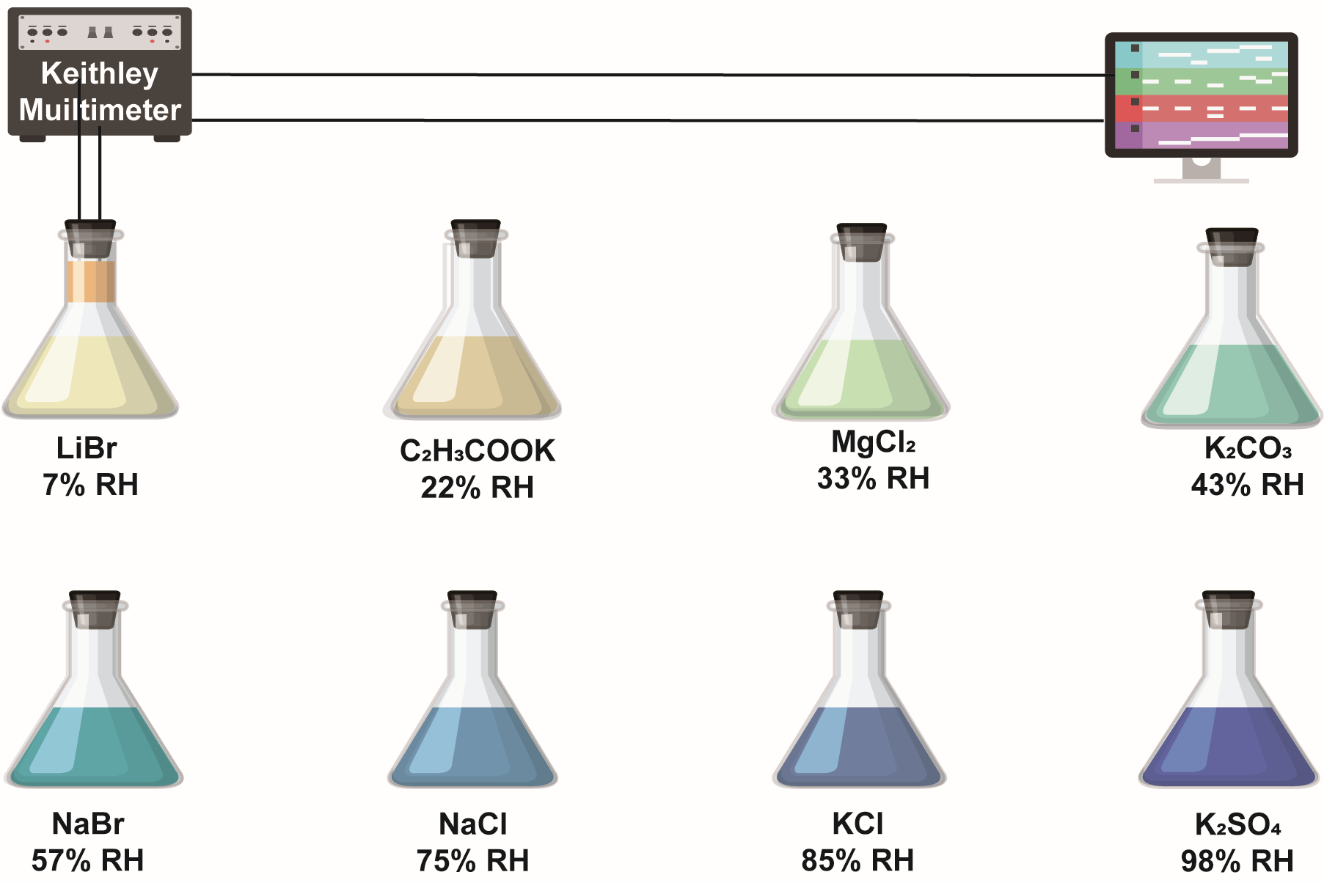


**Figure S6**. Humidity sensor performance test device


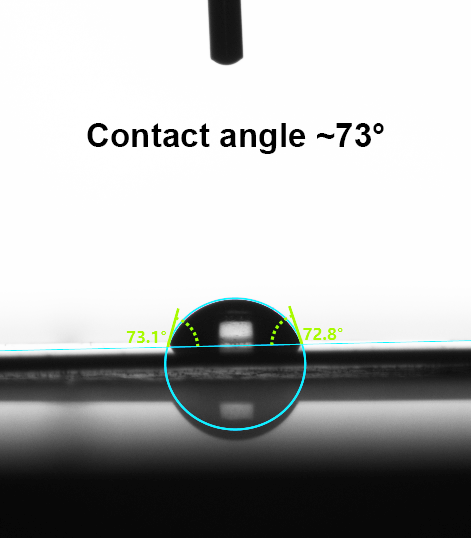


**Figure S7.** Hydrophilicity tests of the AuNPs/PAH


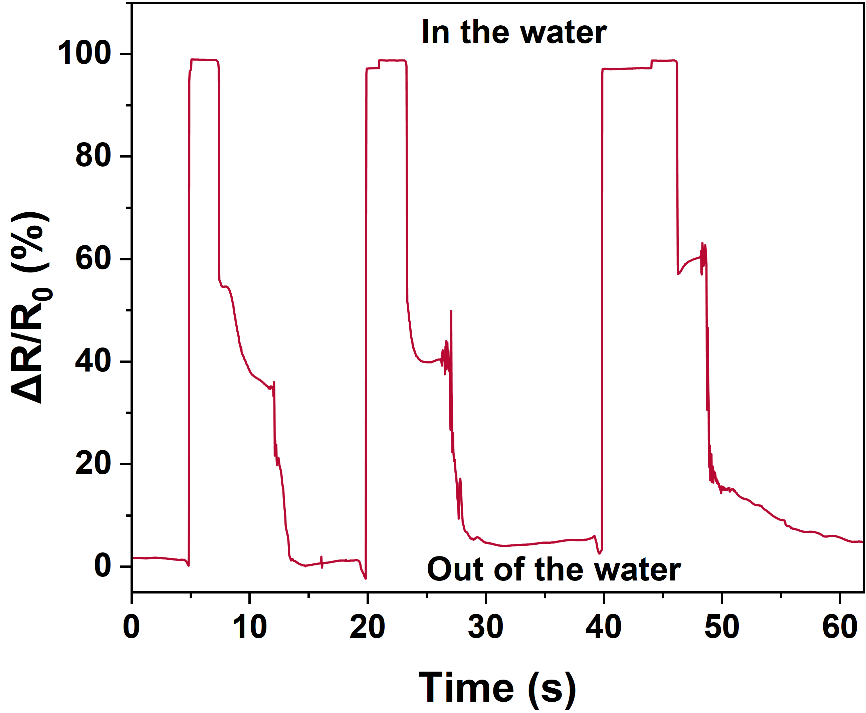


**Figure S8**. The recovery ability of humidity sensors after immersing the water


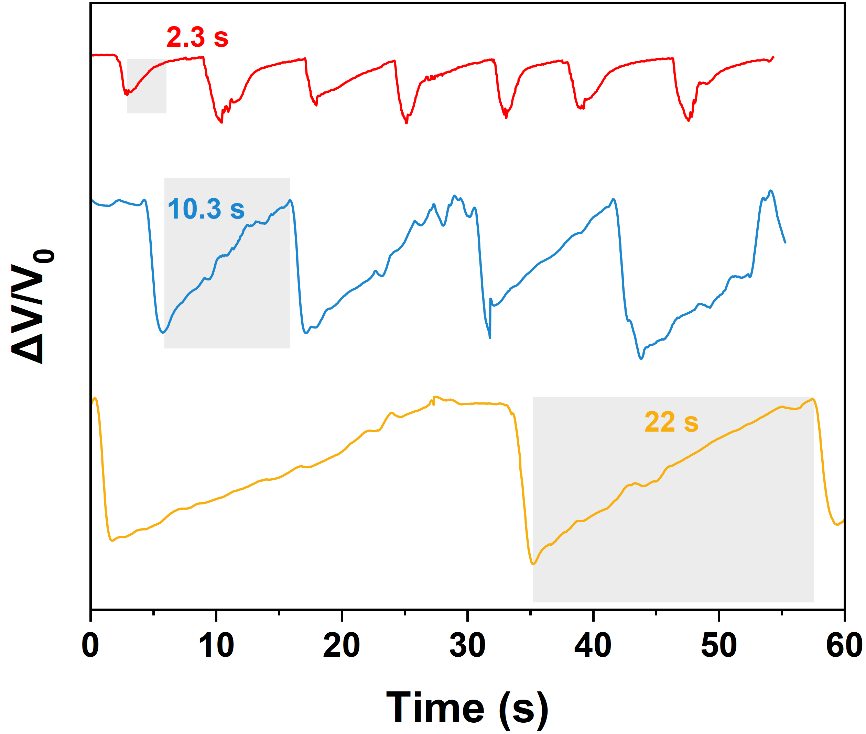


**Figure S9** Sensor response to exhaled gas in different humidity environments


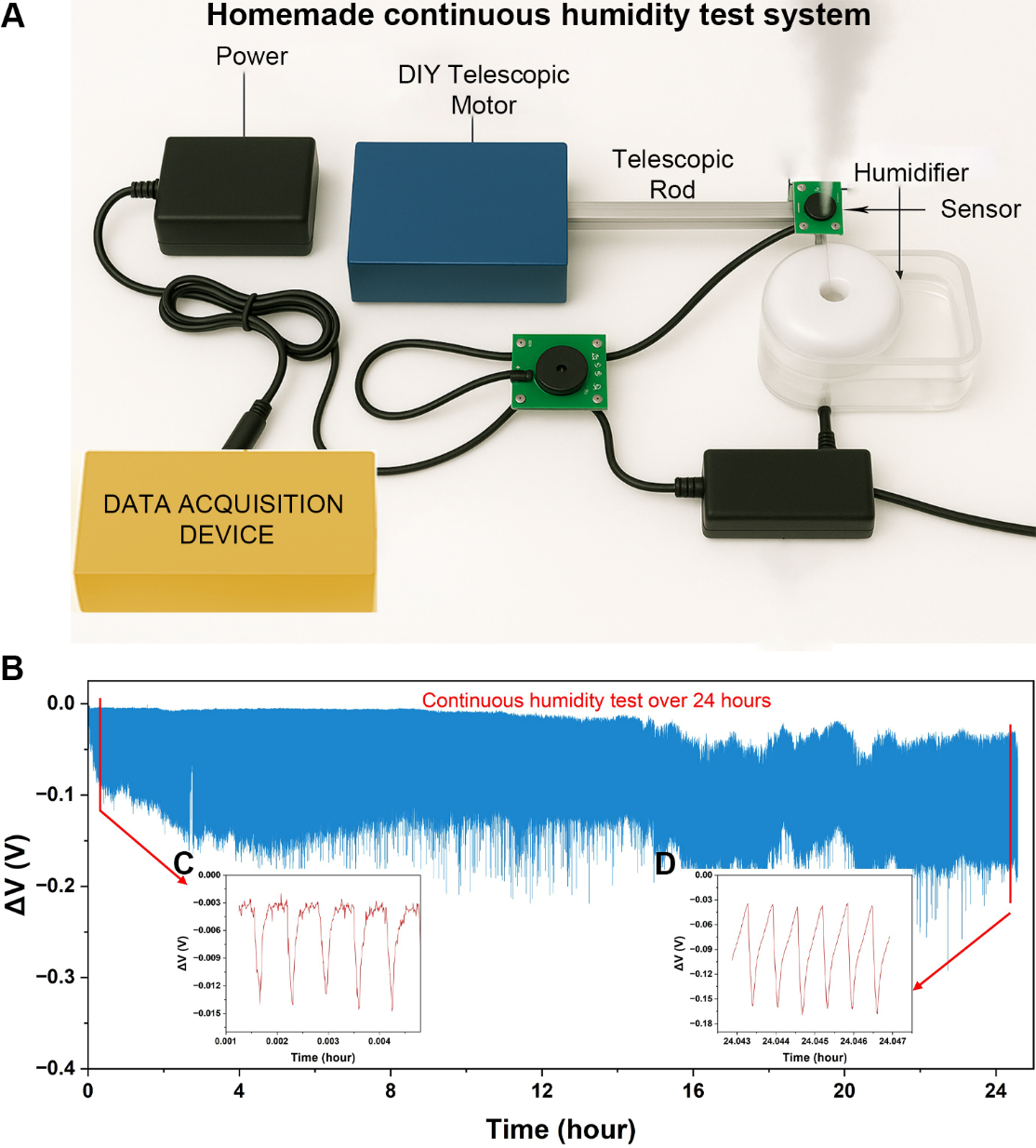


**Figure S10**. Continuous humidity test system. a) The structure of the system. b) Results of continuous testing over 24 hours. c) Response curve of the sensor at the beginning of the experiment. d) Response curve of the sensor after 24 hours


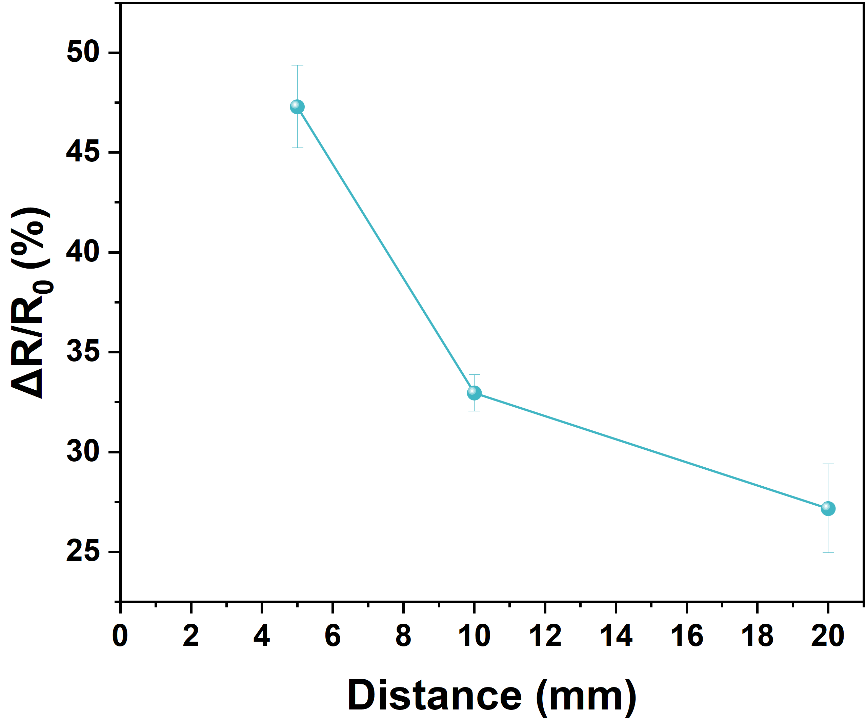


**Figure S11**. Response test of the distance between the sensor and the surface of the water


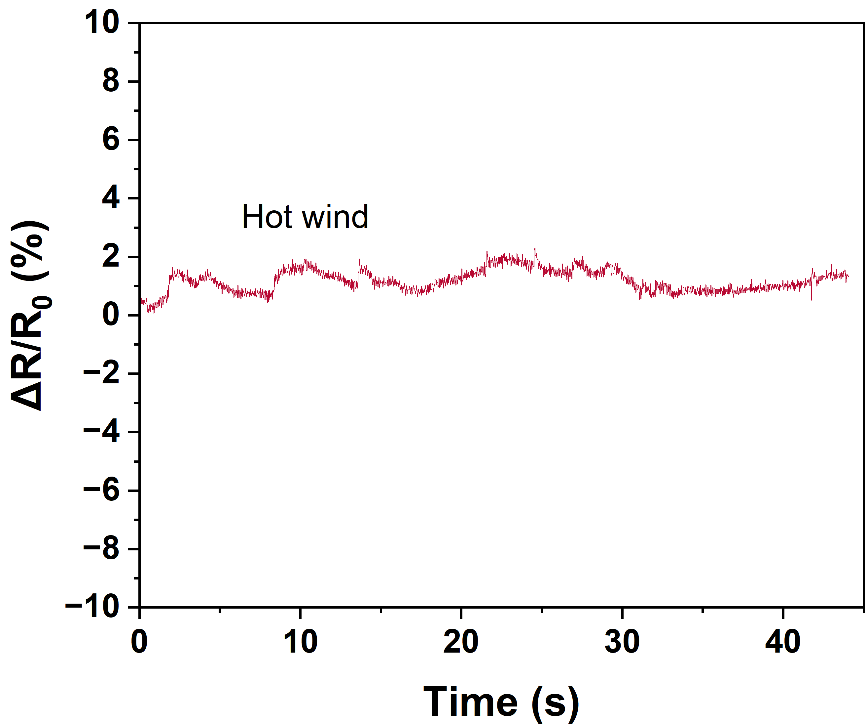


**Figure S12**. The resistance variation of RMHS in the hot wind


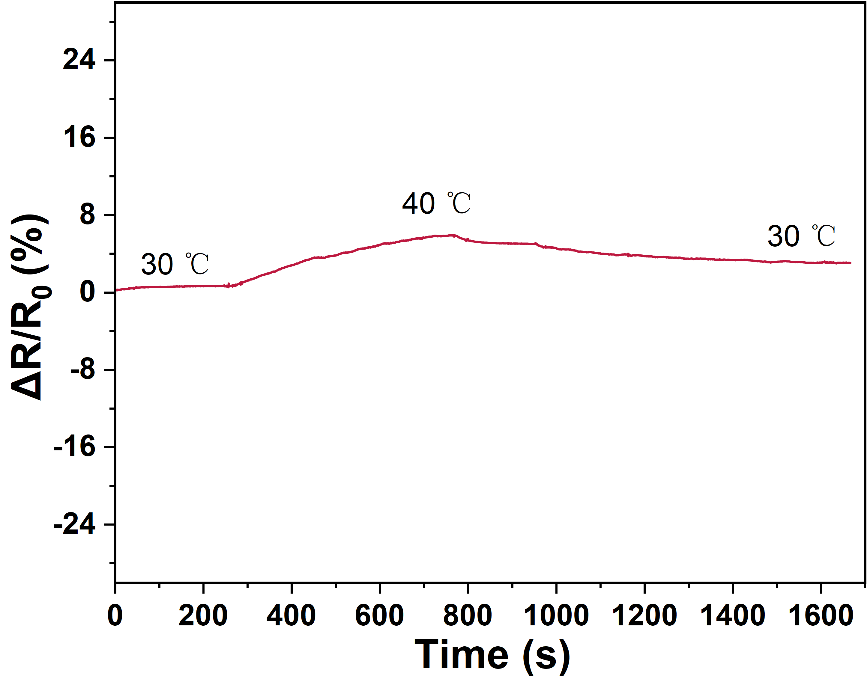


**Figure S13**. The resistance variation of HSRM in the temperature range


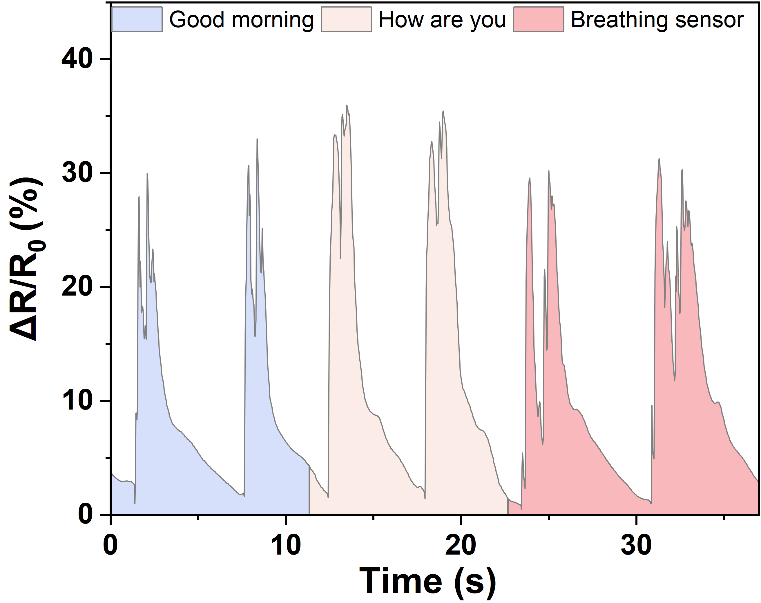


**Figure S14** Detection results of the exhaled respiration language

**CNN algorithm steps:**

The network consists of 49 layers, including convolutional layers, residual Bottleneck blocks, and fully connected layers. The architecture begins with a 7x7 convolutional layer with a stride of 2 and padding of 1, followed by batch normalization and a ReLU activation function. A 3x3 max-pooling layer with a stride of 2 and padding of 1 further reduces the spatial dimensions. The core of the network comprises five sequential layers (layer1 to layer5), each containing a different number of Bottleneck blocks: Layer 1 has 1 block with 16 filters, Layer 2 has 2 blocks with 32 filters, Layer 3 has 4 blocks with 64 filters, and both Layers 4 and 5 have 4 blocks with 128 filters. Each Bottleneck block contains three convolutional layers: a 1x1 convolution to reduce dimensionality, a 3x3 convolution for spatial processing, and a 1x1 convolution to restore dimensionality. Batch normalization and ReLU activation are applied after each convolutional layer, and a residual connection ensures efficient gradient flow. After feature extraction, a 7x7 average pooling layer reduces the spatial dimensions, and the output is passed through two fully connected layers: the first with 512 units and ReLU activation, and the second mapping to the number of output classes. The primary activation function used throughout the network is ReLU, and we employ Kaiming initialization for the convolutional layers. The model outputs class scores, which can be converted to probabilities using a softmax function during evaluation.


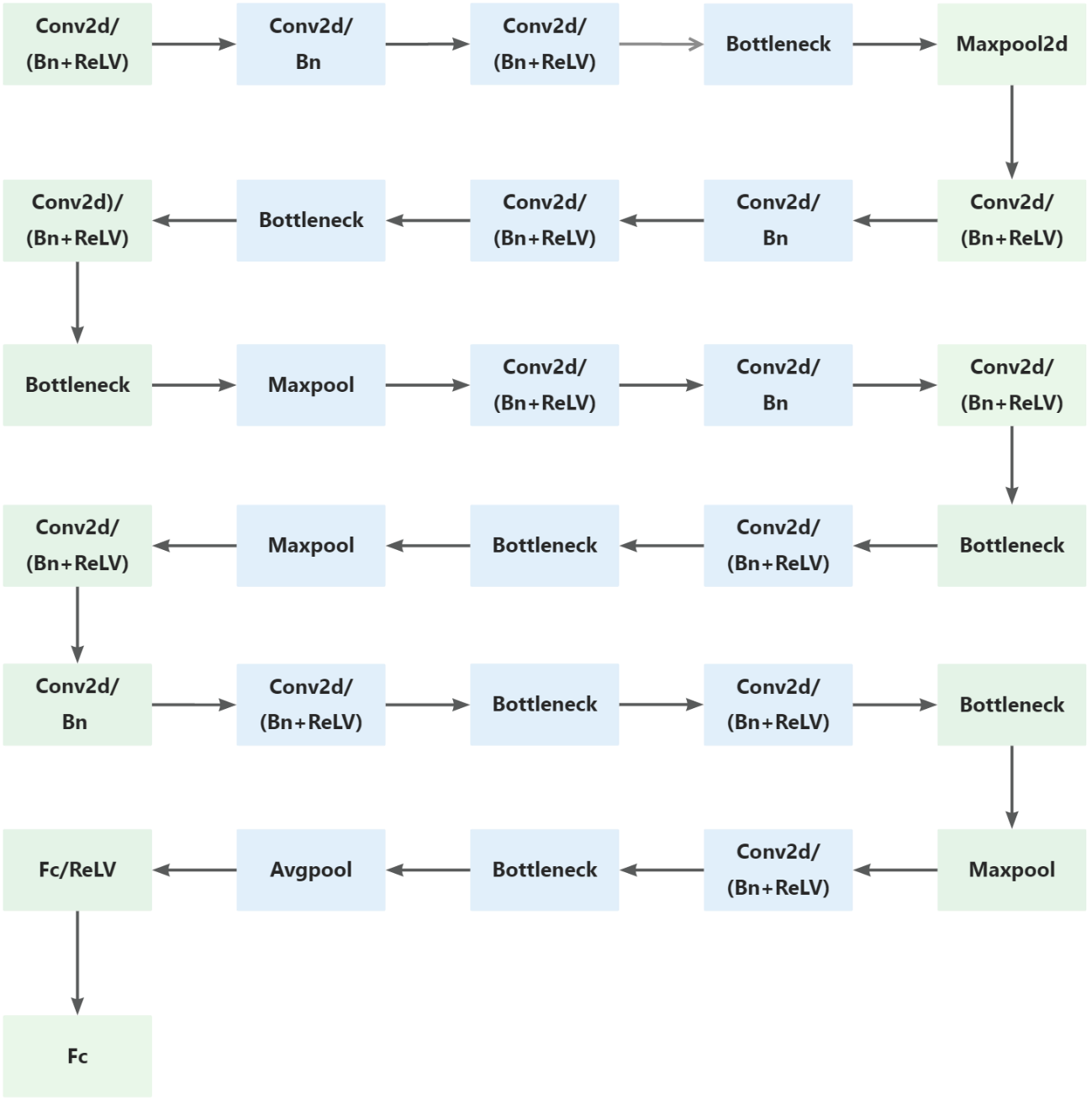


**Figure S15**. CNN algorithm steps for respiration language recognition.

**Reference:**

[1] J. Wu, Y.-M. Sun, Z. Wu, X. Li, N. Wang, K. Tao, G. P. Wang, *ACS Appl. Mater. Interfaces* **2019**, *11*, 4242.

[2] X. Yao, L. Chen, Z. Luo, C. Ye, F. Liang, T. Yang, X. Liu, X. Tian, H. Bi, C. Wang, C. Cai, L. Lyu, X. Wu, *Nano Sel.* **2022**, *3*, 1168.

[3] Y. Liu, X. Li, H. Yang, P. Zhang, P. Wang, Y. Sun, F. Yang, W. Liu, Y. Li, Y. Tian, S. Qian, S. Chen, H. Cheng, X. Wang, *ACS Nano* **2023**, *17*, 5588.

[4] G. Niu, Z. Wang, Y. Xue, J. Yan, A. Dutta, X. Chen, Y. Wang, C. Liu, S. Du, L. Guo, P. Zhou, H. Cheng, L. Yang, *Nano Lett.* **2023**, *23*, 1252.

[5] M. Tang, X. Liu, D. Zhang, H. Zhang, G. Xi, *Sens. Actuators B Chem.* **2024**, *407*, 135429.

[6] L. Chen, K. Hu, M. Lu, Z. Chen, X. Chen, T. Zhou, X. Liu, W. Yin, C. Casiraghi, X. Song, *Adv. Mater.* **2024**, *36*, 2312621.

[7] S. Mondal, S. J. Kim, C.-G. Choi, *ACS Appl. Mater. Interfaces* **2020**, *12*, 17029.

[8] T. Liu, D. Qu, L. Guo, G. Zhou, G. Zhang, T. Du, W. Wu, *Adv. Sens. Res.* **2024**, *3*, 2300014.

[9] X. Li, Y. Guo, J. Meng, X. Li, M. Li, D. Gao, *Langmuir* **2022**, *38*, 8232.

[10] T. Li, T. Zhao, H. Zhang, L. Yuan, C. Cheng, J. Dai, L. Xue, J. Zhou, H. Liu, L. Yin, J. Zhang, *Npj Flex. Electron.* **2024**, *8*, 3.

[11] H. Liu, J. Qin, X. Yang, C. Lv, W. Huang, F. Li, C. Zhang, Y. Wu, L. Dong, C. Shan, *Nano Res.* **2023**, *16*, 10279.

[12] R. A. Shaukat, M. U. Khan, Q. M. Saqib, M. Y. Chougale, J. Kim, A. Bermak, J. Bae, *Sens. Actuators B Chem.* **2022**, *358*, 131507.

[13] N. M. Pereira, N. P. Rezende, T. H. R. Cunha, A. P. M. Barboza, G. G. Silva, D. Lippross, B. R. A. Neves, H. Chacham, A. S. Ferlauto, R. G. Lacerda, *ACS Omega* **2022**, *7*, 9388.

[14] W. Bao, F. Chen, H. Lai, S. Liu, Y. Wang, *Sens. Actuators B Chem.* **2021**, *349*, 130794.

[15] J. Tobin, K. Tomanek, S. Venugopalan, **2024**, DOI 10.48550/arXiv.2412.19315.

[16] G. Anushiya Rachel, N. Sripriya, P. Vijayalakshmi, T. Nagarajan, *Circuits Syst. Signal Process.* **2018**, *37*, 2074.

[17] J. Shor, D. Emanuel, O. Lang, O. Tuval, M. Brenner, J. Cattiau, F. Vieira, M. McNally, T. Charbonneau, M. Nollstadt, A. Hassidim, Y. Matias, in *Interspeech 2019*, **2019**, pp. 784–788.

[18] L. Rose, L. Istanboulian, O. M. Smith, S. Silencieux, B. H. Cuthbertson, A. C. K.-B. Amaral, I. Fraser, J. Grey, C. Dale, *J. Crit. Care* **2018**, *47*, 109.

[19] K. Sato, M. Okajima, T. Taniguchi, *Intensive Care Med.* **2016**, *42*, 1299.

[20] P. R. Tuinman, S. ten Hoorn, Y. J. Aalders, P. W. Elbers, A. R. Girbes, *Intensive Care Med.* **2015**, *41*, 547.
